# BRD4 inhibition leads to MDSC apoptosis and enhances checkpoint blockade therapy

**DOI:** 10.1172/JCI181975

**Published:** 2025-08-05

**Authors:** Himanshu Savardekar, Andrew Stiff, Alvin Liu, Robert Wesolowski, Emily Schwarz, Ian C. Garbarine, Megan C. Duggan, Sara Zelinskas, Jianying Li, Gabriella Lapurga, Alexander Abreo, Lohith Savardekar, Ryan Parker, Julia Sabella, Mallory J. DiVincenzo, Brooke Benner, Steven H. Sun, Dionisia Quiroga, Luke Scarberry, Gang Xin, Anup Dey, Keiko Ozato, Lianbo Yu, Merve Hasanov, Debasish Sundi, Richard C. Wu, Kari L. Kendra, William E. Carson

**Affiliations:** 1 The Ohio State University Comprehensive Cancer Center, Columbus, Ohio, USA.; 2 Biomedical Sciences Graduate Program,; 3 Division of Medical Oncology, Department of Internal Medicine,; 4 Division of Surgical Oncology, Department of Surgery, and; 5 Pelotonia Institute for Immuno-Oncology, The James Comprehensive Cancer Center, The Ohio State University, Columbus, Ohio, USA.; 6 Division of Developmental Biology, National Institute of Child Health and Human Development, Bethesda, Maryland, USA.; 7 Department of Biomedical Informatics and; 8 Division of Urologic Oncology, Department of Urology, The Ohio State University, Columbus, Ohio, USA.

**Keywords:** Immunology, Oncology, Apoptosis, Cancer, Cancer immunotherapy

## Abstract

BRD4 is an epigenetic reader protein that regulates oncogenes such as *myc* in cancer. However, its additional role in shaping immune responses via regulation of inflammatory and myeloid cell responses is not yet fully understood. This work further characterized the multifaceted role of BRD4 in antitumor immunity. Nanostring gene expression analysis of EMT6 tumors treated with a BRD4 inhibitor identified a reduction in myeloid gene expression signatures. Additionally, BRD4 inhibition significantly reduced myeloid-derived suppressor cells (MDSCs) in the spleens and tumors of mice in multiple tumor models and also decreased the release of tumor-derived MDSC growth and chemotactic factors. Pharmacologic inhibition of BRD4 in MDSCs induced apoptosis and modulated expression of apoptosis regulatory proteins. A BRD4 myeloid–specific knockout model suggested that the dominant mechanism of MDSC reduction after BRD4 inhibition was primarily through a direct effect on MDSCs. BRD4 inhibition enhanced anti–PD-L1 therapy in the EMT6, 4T1, and Lewis lung carcinoma tumor models, and the efficacy of the combination treatment was dependent on CD8^+^ T cells and on BRD4 expression in the myeloid compartment. These results identify BRD4 as a regulator of MDSC survival and provide evidence to further investigate BRD4 inhibitors in combination with immune-based therapies.

## Abstract 2

Inhibiting BRD4 can reduce suppressive immune cells. This has the potential to enhance current cancer immunotherapies in the context of solid tumors. .

## Introduction

BRD4 is a member of the bromodomain and extra terminal domain (BET) family of proteins along with BRD2, BRD3, and BRDT (1). Through its function at super-enhancer regions and its direct interaction with transcription factors, BRD4 regulates the expression of several oncogenes such as *MYC* (2, 3). *BRD4* expression is higher in cancers than in normal tissues and is correlated with poor overall survival (4). There has been substantial investigation into the use of BRD4 inhibitors to directly target tumor cells, and several BRD4 inhibitors are being tested in clinical trials (1, 3).

BRD4 also regulates inflammatory responses, and studies have suggested BRD4 plays a role in regulating tumor-associated inflammation and immune responses (5–7). Following oncogene activation, BRD4 redistributes to super-enhancers that promote cytokine production and elimination of premalignant lesions (5). However, in advanced tumors, the role of BRD4 in regulating immune responses is complex and could favor immune evasion (6). BRD4 reduces MHC I expression and antigen processing in the context of cancer, and BRD4 activity may promote tumor progression by increasing chemokine-mediated recruitment of myeloid cells to the tumor (7, 8). BRD4 also increases the ability of macrophages to respond to inflammatory signals and produce cytokines such as IL-6, IL-12, and IL-1β, and it influences the proliferation and survival of immune cells, including those of the myeloid lineage (8–11). Furthermore, BRD4 expression in breast cancer tissues correlates with higher levels of infiltrating myeloid cells (4), and it appears that BRD4 inhibitors may alter the composition of the tumor microenvironment (TME) (12–16). However, the mechanism by which BRD4 inhibition leads to these effects is unclear.

Myeloid-derived suppressor cells (MDSCs) are a component of the TME with immune-suppressive functions that promote carcinogenesis and disease progression (17, 18). These functions include the production of immunosuppressive cytokines (TGF-β and IL-10) and reactive nitrogen and oxygen species (NO, ROS, and PNT) and depletion of key nutrients from the TME that are required by T cells and NK cells (l-arginine, l-tryptophan, and l-cysteine) (19–21). MDSCs can be divided into 2 major subsets, polymorphonuclear (PMN-MDSCs) and monocytic (M-MDSCs), with the latter (M-MDSCs) being able to differentiate into tumor-associated macrophages (TAMs) after migration into the TME (22). Targeting these tumor-infiltrating myeloid cells can result in reduced tumor growth and improved survival in preclinical models and has the potential to enhance the immune-based cancer therapy (23–25). Preliminary mathematical modeling of BRD4 inhibition with a CTLA-4 blockade suggested combinatorial efficacy between these 2 agents, prompting our further investigation into its therapeutic potential and mechanism of action (14).

Our group hypothesized that BRD4 inhibition would impact the composition of the TME primarily by impacting the myeloid compartment. We found that systemic inhibition of BRD4 decreased levels of MDSCs in the spleens and tumors of mice. Using a myeloid-specific BRD4-knockout mouse model (*Brd4^fl/fl^ LysM-Cre*), myeloid lineage loss of BRD4 decreased splenic and tumor MDSCs in the Lewis lung carcinoma (LLC) model. BRD4 inhibition decreased MDSC survival and was associated with modulation of apoptotic protein expression. Finally, in multiple tumor models, BRD4 inhibition improved the efficacy of anti–PD-L1 therapy. To our knowledge, this is the first report to demonstrate the role of BRD4 in MDSC survival and propose an immunologic mechanism behind the enhancement of immune checkpoint therapy by BRD4 inhibition. These findings provide a rationale for the utilization of BRD4 inhibitors in combination with immune-based treatments in the clinical setting.

## Results

### Nanostring tumor gene profiling reveals decreased myeloid cell infiltration after BRD4 inhibition.

**Figure 1:**
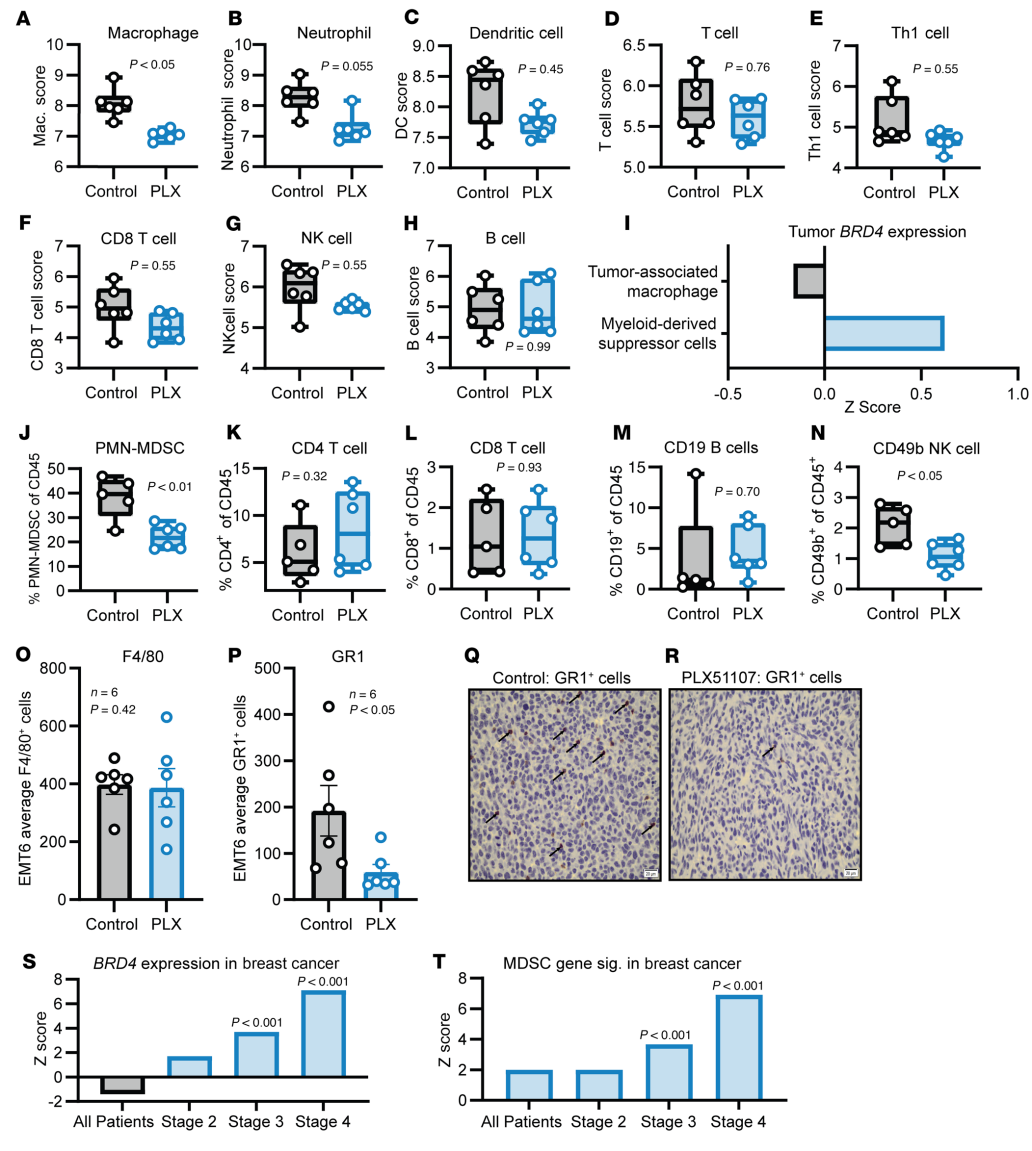
Nanostring pan-cancer immune profiling identifies reduced myeloid cell infiltration of EMT6 tumors treated with PLX51107. (**A**–**H**) BALB/c mice were inoculated with 1 × 10^6^ EMT6 cells and treated with vehicle (control) or 20 mg/kg PLX51107 (PLX) daily p.o. once tumors were palpable (50 mm^3^). After 1 week of treatment, RNA was extracted from whole tumors. Gene expression was then analyzed using the nCounter PanCancer Immune Profiling Panel. Raw cell abundance scores for indicated cell types are displayed on a logarithmic scale. A difference in the mean raw abundance score of 1 between control and PLX51107 treatments indicates a 2-fold difference. ANOVA models and *t* statistics were used for the comparison of cell type scores (log_2_) between control and PLX51107. (**I**) Pan-cancer BRD4 tumor gene expression association with gene signatures of suppressive cell types. Expression signatures were part of the TIDE algorithm. *z* > 0 represents a positive association. (**J**–**N**) BALB/c mice were inoculated with 1 × 10^5^ 4T1 cells and treated with vehicle (control) or 20 mg/kg PLX51107 daily p.o. once tumors were palpable (50 mm^3^). After 8 days of treatment, tumors were processed into single-cell suspensions and stained with fluorescent antibodies. Cell populations were acquired by spectral flow cytometry, unmixed on the Cytek Aurora 5L cytometer, and processed using the OMIQ software platform. (**O** and **P**) EMT6 tumors from mice treated as in **A** were fixed and stained with an antibody against F4/80 (**O**) or GR1 (**P**) and visualized using an HRP-conjugated secondary antibody. ImageJ software was used to count HRP^+^ cells from 5 high-powered fields per slide to obtain an average number of positive cells. The bar graph represents the mean ± SEM of GR1^+^ cells from 6 slides per treatment group; *P* < 0.05 (unpaired 2-tailed Student’s *t* test). (**Q** and **R**) Representative images of GR1^+^ cells labeled with black arrows in tumors of control (**P**) or PLX51107-treated (**O**) EMT6 tumor–bearing mice. Scale bar: 20 μm. (**S** and **T**) TIMER2 analysis of tumor *BRD4* expression (**S**) or tumor MDSC gene signature expression (**T**) in The Cancer Genome Atlas breast cancer patients at different stages (*n* = 1,100). Values on the bar graph are *z* scores with the Cox proportional hazard model to evaluate significance. *z* > 0 indicates a positive association. MDSC gene signature expression was quantified using the TIDE algorithm.

The nCounter PanCancer Immune Profiling Panel was used to explore differences in the TME between mice bearing EMT6 tumors (murine triple-negative breast cancer model) (26) that were treated with a vehicle control or the BET inhibitor PLX51107. PLX51107 is a potent and selective BET inhibitor targeting the bromodomains of BRD4, BRD2, and BRD3 and has been widely studied as a BRD4 inhibitor (27). Cell type profiling showed that PLX51107 altered myeloid populations with a reduction in the abundance scores of macrophages and neutrophils (Figure 1, A and B; *P* < 0.05 for macrophages, *P* = 0.055 for neutrophils). The abundance scores for other cell types (dendritic cells, T cell subsets, NK cells, B cells, and CD45 cells) trended lower in the PLX51107 group, but the differences were low in magnitude and not statistically significant with the exception of NK CD56^dim^ cells (Figure 1, C–H, and Supplemental Figure 1, A–D; supplemental material available online with this article; https://doi.org/10.1172/JCI181975DS1). These results suggested that the intratumoral myeloid populations were most affected by BRD4 inhibition.

To further explore the association between tumor *BRD4* expression and myeloid cell populations, the TIDE (tumor immune dysfunction and exclusion) tool was employed (28). Tumor *BRD4* gene expression was quantified using RNA-Seq across pan-cancer datasets from 189 human cancer studies by the Liu group (28). This analysis showed a positive association (*z* score) with MDSC gene signature but a negative association with TAM gene signature (Figure 1I). To expand upon this finding, changes in intratumoral cell populations after PLX51107 treatment were analyzed via spectral flow cytometry in the 4T1 triple-negative breast cancer tumor model. Similar findings were observed with a significant reduction in PMN-MDSCs, while other non-myeloid populations remained similar with the exception of CD49b^+^ NK cells (*n* = 5–6, *P* < 0.05) (Figure 1, J–N, and Supplemental Figure 1, E–I). Moreover, IHC for F4/80 and GR1 was performed on control and PLX51107-treated EMT6 tumors. No significant changes in the number of F4/80^+^ cells per 5 high-power levels were detected, but a significant decrease in GR1^+^ cells was observed (Figure 1, O–R).

The TIMER2 (tumor immune estimation resource) and TIDE tools were used to explore the association between *BRD4* gene expression and MDSC populations in tumors from human breast cancer patients (28). A positive association (*z* score) was found between breast cancer *BRD4* expression and clinical breast cancer stage (stages 3 and 4, *P* < 0.001) (Figure 1S). Breast cancer stage was also positively associated with MDSC gene expression signature (stages 3 and 4, *P* < 0.001) (Figure 1T).

### BRD4 inhibition reduces MDSCs in multiple tumor models.

**Figure 2:**
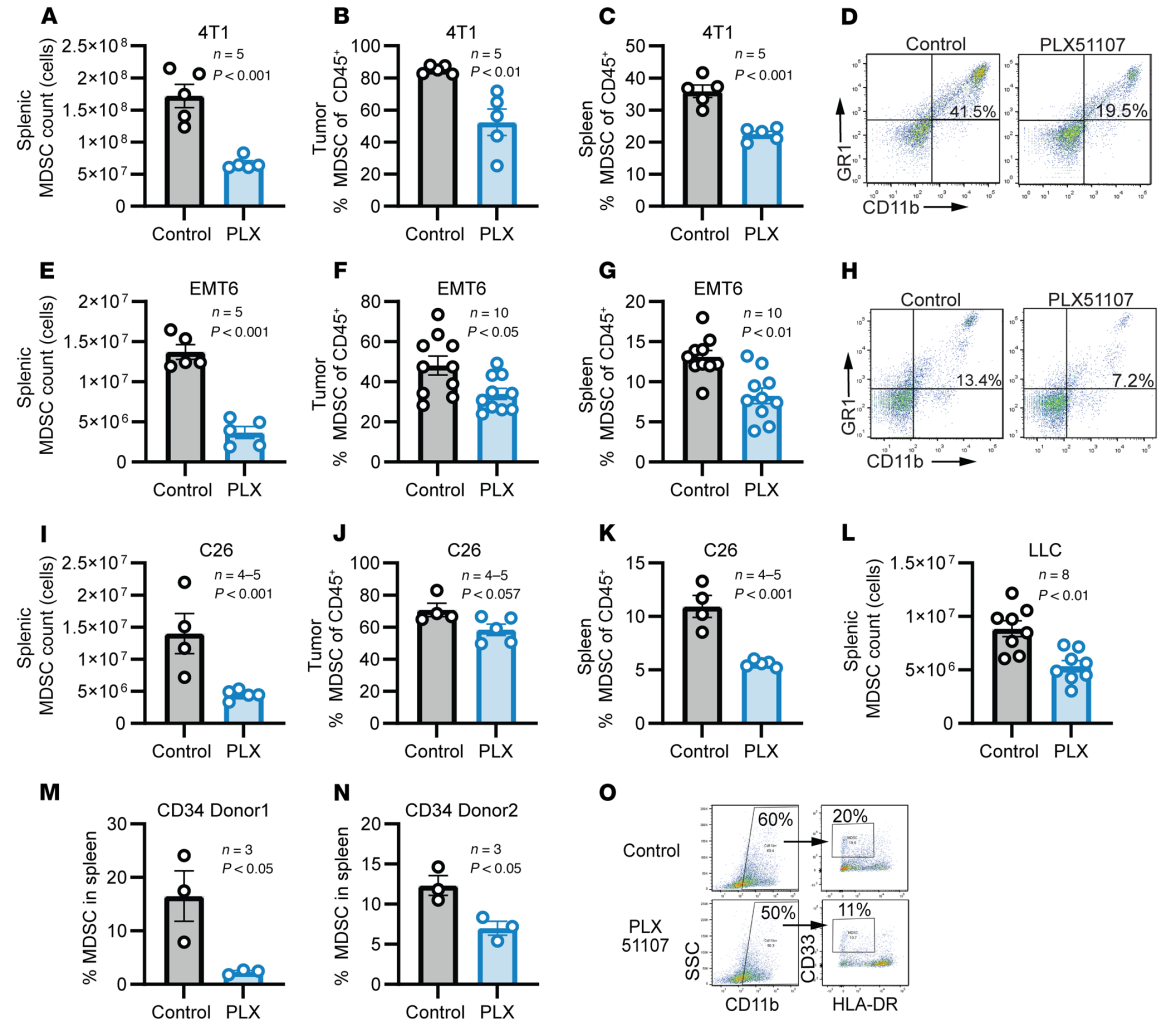
PLX51107 reduces MDSCs in vivo. (**A**–**D**) The 4T1 tumor–bearing mice were treated with vehicle (control) or 20 mg/kg PLX51107 (PLX) daily once tumors reached 50 mm^3^ for 9 days. Absolute MDSC count was calculated by multiplying total splenocyte number (counted using a Z-Series Coulter Counter) by MDSC frequency as measured by flow cytometry. (**A**) Values represent mean ± SEM of absolute MDSC count (*n* = 5); *P* < 0.001, unpaired 2-tailed Student’s *t* test. (**B** and **C**) Values represent mean ± SEM of frequency of CD45^+^/GR1^+^/CD11b^+^ MDSCs in (**B**) tumor and (**C**) spleen (*n* = 5); *P* < 0.01 and *P* < 0.001 for MDSCs within the tumor and spleen, respectively (unpaired 2-tailed Student’s *t* test). (**D**) Representative flow cytometry plots of MDSCs in the spleen. (**E**–**H**) EMT6 tumor–bearing mice were treated as in **A**. (**E**) Values represent mean ± SEM of MDSC counts (*n* = 5); *P* < 0.0001, unpaired 2-tailed Student’s *t* test. (**F** and **G**) Values represent mean ± SEM of frequency of MDSCs in (**F**) tumor and (**G**) spleen (*n* = 10); *P* < 0.05 and *P* < 0.01 for MDSCs within the tumor and spleen, respectively, unpaired 2-tailed Student’s *t* test. (**H**) Representative flow cytometry plots of MDSCs in the spleen. (**I**–**K**) C26 tumor–bearing mice were treated as in **A**. (**I**) Values represent mean ± SEM of splenic MDSC counts (*n* = 4–5); *P* < 0.001, unpaired 2-tailed Student’s *t* test. (**J** and **K**) Frequency of MDSCs of C26 in tumor (**J**) and spleen (**K**). Values represent mean ± SEM of frequency of MDSCs (*n* = 4–5); *P* = 0.057 and *P* < 0.001 for MDSCs within the tumor and spleen, respectively, unpaired 2-tailed Student’s *t* test. (**L**) LLC tumor–bearing mice treated as in **A**. Values represent mean ± SEM of splenic MDSC counts (*n* = 8); *P* < 0.01, unpaired 2-tailed Student’s *t* test. (**M** and **N**) CD34^+^ HSC-engrafted NSG-SGM3 mice were co-engrafted with the melanoma PDX tumor model and treated as in **A** for 15 days. Values represent mean ± SEM of splenic MDSCs (CD11b^+^/CD33^+^/HLA-DR^lo/–^) for each donor (*n* = 3/donor); *P* < 0.05, unpaired 2-tailed Student’s *t* test. (**O**) Representative flow cytometry plots in **M** and **N**.

To further assess the impact of BRD4 inhibition on MDSCs in triple-negative breast cancer (TNBC), the EMT6 and 4T1 breast cancer models were employed. PLX51107 treatment led to a slight reduction of tumor volumes in both models, but was statistically significant only in the 4T1 tumor model (*P* < 0.01) (Supplemental Figure 2, A and D). However, in both models, PLX51107 treatment significantly reduced the number of total splenocytes, the absolute number of MDSCs present in the spleen (4T1, 17.0 × 10^6^ vs. 6.0 × 10^6^; EMT6, 13.0 × 10^6^ vs. 4.0 × 10^6^; *P* < 0.001 for both comparisons), and the percentage of GR1^+^CD11b^+^ MDSCs within the spleen and tumor at the 15 day time point (Figure 2, A–H, and Supplemental Figure 2, E and F; *P* < 0.05, *P* < 0.01, or *P* < 0.001 for all comparisons).

To determine if the reduction in total MDSCs was specific to a subset of MDSCs (PMN-MDSCs or M-MDSCs), the EMT6 model was employed. PLX51107 treatment led to a significant reduction in the PMN-MDSC subset (%CD11b^+^Ly6G^hi^Ly6C^lo^ of CD45^+^ splenocytes 7.4% vs. 3.6%, *P* < 0.01), while there was a nonsignificant 25% decrease in the M-MDSC subset (%CD11b^+^Ly6G^lo^Ly6C^hi^ of CD45^+^ splenocytes 1.37% vs. 1.04%, *P* = 0.33) (Supplemental Figure 2, G–I). In contrast, PLX51107 treatment in the 4T1 model significantly reduced both PMN-MDSCs (19.5% vs. 10.6%, *P* < 0.001) and M-MDSCs (2.8% vs. 1.0%, *P* < 0.001) (Supplemental Figure 2, J–L)

To determine if these findings could be reproduced in non–breast cancer models, the C26 colorectal cancer and LLC models were employed. In both models, PLX51107 treatment reduced the total number of MDSCs within the spleen (C26, 13.9 × 10^6^ vs. 4.5 × 10^6^, *P* < 0.001; LLC, 8.8 × 10^6^ vs. 5.3 × 10^6^, *P* < 0.01), frequency of intratumoral MDSCs (C26, 70.7% vs. 58.3%, *P* = 0.057; LLC, 58.7% vs. 46.0%, *P* < 0.05), and frequency of splenic MDSCs (C26, 10.9% vs. 5.6%, *P* < 0.001; LLC, 6.9% vs. 4.9%, *P* < 0.01), while not significantly affecting tumor weight (Figure 2, I–L, and Supplemental Figure 3, A–D). Similar results were obtained for the PMN-MDSC and M-MDSC subsets in the C26 model (Supplemental Figure 3, E–G). The depletion of MDSCs with BRD4 inhibition was also replicated using 2 other BRD4 inhibitors, PLX2853 and JQ1. PLX2853 significantly reduced spleen weights and MDSCs within the spleen of 4T1 tumor–bearing mice white not significantly affecting tumor volumes and weight (%MDSCs in spleen: 30.1% vs. 18.9%, *P* < 0.05) (Supplemental Figure 4, A–E). In the EMT6 model, JQ1 significantly reduced splenic and intratumoral MDSCs while not significantly affecting tumor volumes (Supplemental Figure 4, F–I; tumor, *P* < 0.001; spleen, *P* < 0.05).

To explore if these findings could be recapitulated in humans, a humanized mouse model was employed. CD34^+^ hematopoietic stem cell–engrafted (HSC-engrafted) NOD-*scid* IL2Rg^null^ (NSG-GM3) mice were co-engrafted with syngeneic human melanoma tumors using the melanoma patient-derived xenograft (PDX) tumor model TM01149 (The Jackson Laboratory). After 10 days of treatment, PLX51107 reduced tumor volumes, and this was significant in 1 donor (Supplemental Figure 5, A and B). BRD4 inhibition also decreased the size of the spleen and the percentage of splenic MDSCs (CD11b^+^CD33^+^HLA-DR^lo/–^) in CD34^+^ donor 1 (16.4%–2.4%, *P* < 0.05) and CD34^+^ donor 2 (12.3%–7.9%, *P* < 0.05) (Figure 2, M–O, and Supplemental Figure 5, C and D). IHC analysis of the tumors revealed a reduction in CD206^+^ cells, a marker of TAMs known to differentiate from M-MDSCs (30–32), in 1 donor with PLX51107 treatment (*P* < 0.05) (Supplemental Figure 5E).

In all models, BRD4 inhibition reduced tumor volumes, and in the 4T1 model treated with PLX51107, this effect reached statistical significance, suggesting that the reductions in MDSC numbers could be due to reduced tumor burden (Supplemental Figure 2A). To address this possibility, advanced 4T1 tumors (treatment started 2 weeks after inoculation) were treated with PLX51107 for just 5 days. In these experiments, tumor weights between treatment groups were essentially the same (Supplemental Figure 6A; *P* = 0.89), but PLX51107 significantly reduced the frequency of total MDSCs (Supplemental Figure 6B; 28.7% vs. 17.9%, *P* < 0.05), PMN-MDSCs (Supplemental Figure 6C; 17.9% vs. 8.4%, *P* < 0.001), and M-MDSCs (Supplemental Figure 6C; 1.8% vs. 1.1%, *P* < 0.01) in the spleen. These findings were similarly significant with JQ1 treatment as well (Supplemental Figure 6, D–F).

### BRD4 deficiency in the myeloid compartment results in decreased MDSC frequency.

**Figure 3:**
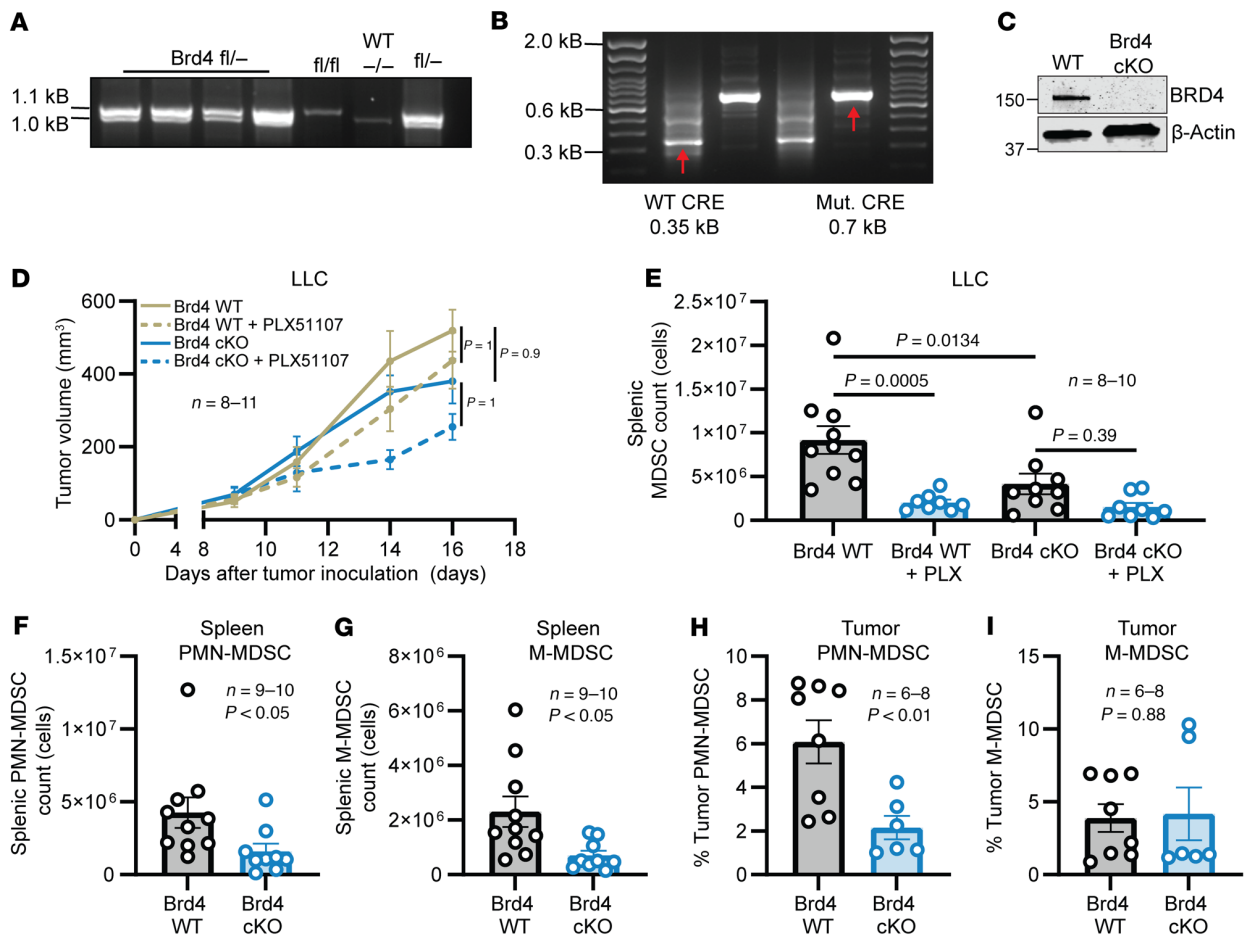
Loss of BRD4 in the myeloid compartment results in decreased MDSC frequency. (**A**) Genotyping of *Brd4^fl/fl^ LysM-Cre* mice. Presence of BRD4^fl/fl^ allele at 1.1 kB and WT allele at 1.0 kB. (**B**) Presence of mutant Cre expression of BRD4^fl/fl^ mice at 700 bp (Cre^+^) and WT Cre allele at 350 bp (Cre^–^). (**C**) Immunoblot of BRD4 expression in splenic MDSCs of *Brd4^fl/fl^ LysM-Cre*^+^ (BRD4 cKO) and littermate control (BRD4 WT) mice implanted with LLC tumors. (**D**) BRD4 cKO and WT mice inoculated with 1 × 10^6^ LCC cells subcutaneously and treated with the vehicle control or 20 mg/kg PLX51107 daily via oral gavage once tumors were palpable (50 mm^3^) for 8 days. Tumor volumes were measured 3 times weekly with digital calipers. Values are the mean ± SEM of tumor volumes at each time point (*n* = 8–11); *P* > 0.05 for all groups, linear mixed model with Tukey-Kramer adjustment. (**E**) Absolute total MDSC (CD11b^+^/GR1^+^) count within the spleen of BRD4 WT and BRD4 cKO LLC tumor–bearing mice treated with the vehicle control or 20 mg/kg PLX51107 (PLX). Absolute MDSC counts were calculated as above. Values represent mean ± SEM from 8–10 mice per treatment group. One-way ANOVA model with Tukey’s correction. (**F** and **G**) Absolute counts of MDSC subsets (**F**; PMN-MDSC) and (**G**; M-MDSC) within the spleen of BRD4 WT and BRD4 cKO LLC tumor–bearing mice on day 16 after tumor implantation. Absolute MDSC counts were calculated as in Figure 1A. Values represent mean ± SEM from 9–10 mice; *P* < 0.05, unpaired 2-tailed Student’s *t* test. (**H** and **I**) Frequency of PMN-MDSCs (CD11b^+^, Ly6G^+^, Ly6C^mid^) and M-MDSCs (CD11b^+^, Ly6^–^, Ly6C^hi^) within LLC tumors (day 16) of BRD4 WT or BRD4 cKO mice as measured by flow cytometry. Values represent mean ± SEM from 6–8 mice per group; *P* < 0.01 for PMN-MDSCs, unpaired 2-tailed Student’s *t* test.

To determine the intrinsic role of BRD4 in MDSCs, BRD4 was conditionally deleted in the myeloid lineage by breeding the *Brd4^fl/fl^* mice with transgenic *LysM-Cre* mice, which express Cre recombinase under the control of the myeloid cell *Lyz2* promoter. As previously reported, the presence of a *Brd4*-floxed allele at 1.1 kb was confirmed in this model (Figure 3A). *LysM-Cre* expression in *Brd4^fl/fl^* mice was confirmed, and these mice comprised a BRD4 cKO group (Figure 3B). Splenic MDSCs were isolated from LLC tumor–bearing BRD4 cKO mice and littermate controls (WT) to confirm reduced expression of BRD4 by immunoblot (Figure 3C). These assays confirmed the absence of BRD4 in MDSCs obtained from the BRD4 cKO model.

BRD4 cKO and WT mice bearing LLC tumors were treated for 8 days with vehicle or PLX51107. After treatment, there was a nonsignificant reduction of tumor volumes in both BRD4 WT and BRD4 cKO mice (Figure 3D). PLX51107 significantly reduced the absolute count of MDSCs in the spleen of WT mice (9.1 × 10^6^ vs. 2.0 × 10^6^, *P* < 0.001), but the reduction of MDSCs in BRD4 cKO mice receiving PLX51107 did not achieve significance (4.22 × 10^6^ vs. 1.53 × 10^6^, *P* = 0.39) (Figure 3E). Furthermore, BRD4 cKO mice had significantly lower baseline levels of MDSCs compared with BRD4 WT mice (9.17 × 10^6^ vs. 4.22 × 10^6^, *P* = 0.013) (Figure 3E). Additionally, LLC tumor–bearing BRD4 cKO mice had significantly lower baseline levels of both PMN-MDSCs and M-MDSCs in the spleen compared with WT mice (Figure 3, F and G). Only PMN-MDSCs were significantly lower in the tumors of BRD4 cKO mice compared with BRD4 WT mice (Figure 3, H and I). Splenic total numbers were used to indicate the total peripheral burden of MDSCs, and using percent MDSCs in tumors helped to eliminate variability due to tumor size. Intratumoral myeloid populations in the BRD4 cKO mice were analyzed by flow cytometry to determine effects of BRD4 cKO on other myeloid lineages. There were no significant differences in the proportion of TAMs, CD206^–^ M1–like macrophages, or dendritic cells compared with WT mice (Supplemental Figure 6, G–J). Together, the above results suggest that the specific loss of BRD4 in the myeloid compartment impairs the frequency of MDSC accumulation in the setting of malignancy.

### BRD4 inhibition induces MDSC apoptosis.

**Figure 4:**
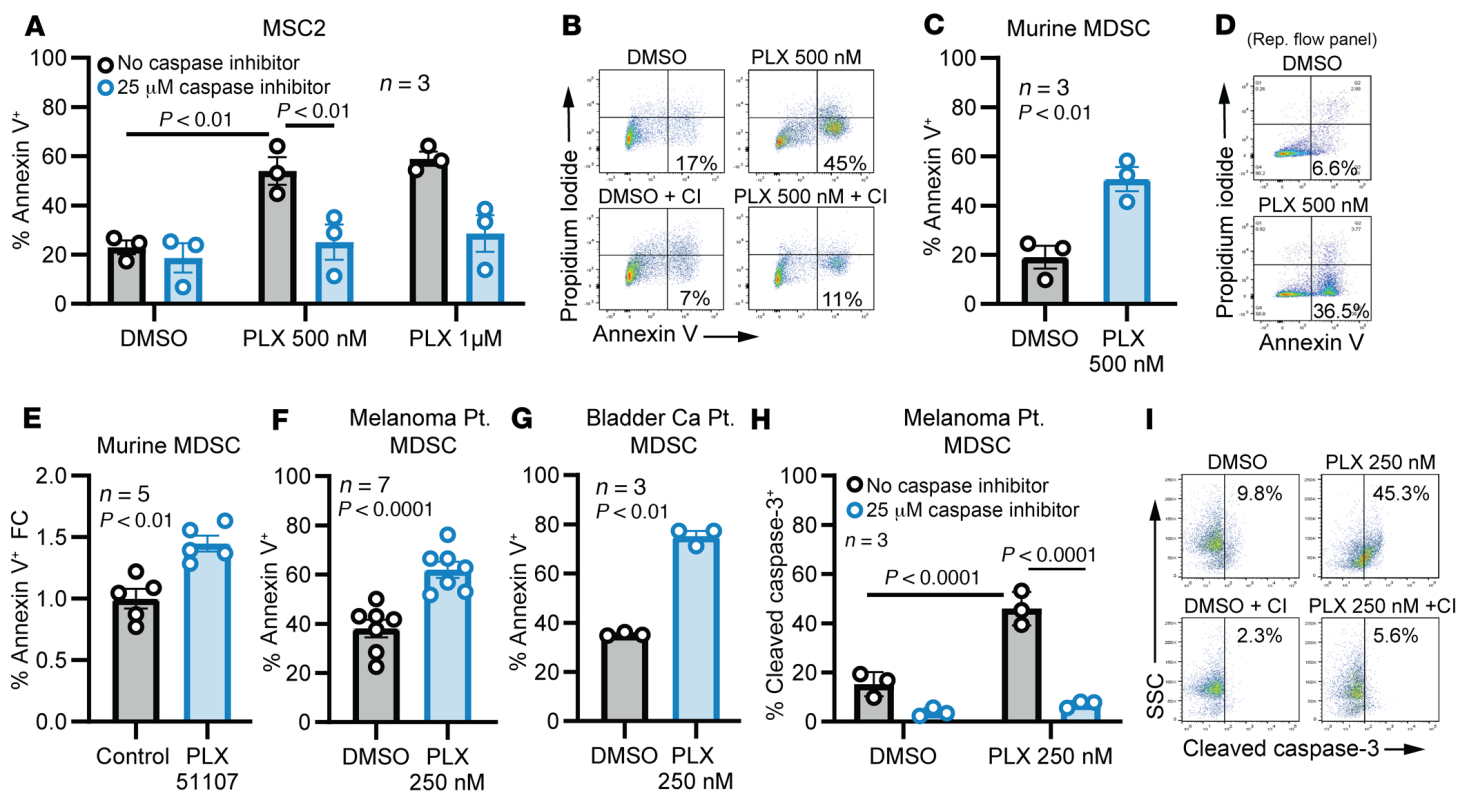
PLX51107 induces MDSC apoptosis. (**A**) Values represent the mean ± SEM of annexin V^+^ MSC2 cells treated as indicated for 24 h (*n* = 3); *P* < 0.01 for DMSO versus 500 nM PLX51107 (PLX), *P* < 0.01 for 500 nM PLX51107 versus 500 nM PLX51107 + 25 μM Z-VAD-FMK, 2-way ANOVA model with Tukey’s correction. (**B**) Representative flow cytometry plot of staining from **A**. Annexin V^+^ and propidium iodide^–^ negative early apoptosis of PLX51107-treated cells is demonstrated. (**C**) Murine splenic MDSCs isolated from 4T1 tumor–bearing mice treated ex vivo with media supplemented with 10 ng/mL IL-6 and GM-CSF for 48 h. Values represent the mean ± SEM of annexin V^+^ murine splenic MDSCs treated as indicated (*n* = 3); *P* < 0.01 for DMSO versus 500 nM PLX51107, unpaired 2-tailed Student’s *t* test. (**D**) Representative flow cytometry plot of staining from **C**. (**E**) 4T1 tumor–bearing mice were treated with control or PLX51107 (20 mg/kg) for 7 days. Splenocytes were isolated and stained for MDSCs (GR1^+^/CD11b^+^) and annexin V. Values represent fold increase of annexin V^+^ MDSCs in mice receiving PLX51107 compared with mice receiving control (*n* = 5); *P* < 0.01, unpaired 2-tailed Student’s *t* test. (**F** and **G**) CD33^+^/CD11b^+^/HLA-DR^lo/–^ MDSCs were isolated from the peripheral blood of patients with melanoma (**F**) or bladder cancer (**G**) by FACS. Cells were cultured in human AB serum media supplemented with 10 ng/mL IL-6 and GM-CSF and treated with DMSO or 250 nM PLX51107 for 48 h. Values represent mean ± SEM of annexin V^+^ cells from experiments; *P* < 0.0001 for DMSO versus 250 nM PLX51107 in **F** (*n* = 7), and *P* < 0.01 for DMSO versus 250 nM PLX51107 in **G** (*n* = 3), paired 2-tailed Student’s *t* test. (**H**) Values represent mean ± SEM of cleaved caspase-3^+^ melanoma patient MDSCs isolated as in **F** and treated as indicated for 48 h (*n* = 3); *P* < 0.0001 for DMSO versus 250 nM PLX51107 and 250 nM PLX51107 versus 250 nM PLX51107 + 25 μM Z-VAD-FMK, 2-way ANOVA model with Tukey’s correction. (**I**) Representative flow cytometry plot of staining from **H**.

BRD4 is known to play a role in regulating survival and apoptosis in several cell types (33, 34). To determine if BRD4 inhibition could induce apoptosis of MDSCs, the murine MDSC cell line MSC2 was initially employed (35). Treatment of MSC2 cells with PLX51107 or JQ1 for 24 h resulted in a dose-dependent induction of apoptosis, and this effect was reversed by the pan-caspase inhibitor Z-VAD-FMK (Figure 4, A and B, and Supplemental Figure 7A; *P* < 0.01 for all comparisons). PLX51107 also induced strong activation of caspase-3, as measured by a fluorometric assay using MSC2 cells (Supplemental Figure 7B; *P* < 0.01). In splenic MDSCs isolated from 4T1 tumor–bearing mice, PLX51107 significantly reduced Ki67 positivity at 72 h with a sublethal dose of 62.5 nM (50% vs. 34%, *P* < 0.05) (Supplemental Figure 7, C and D). A greater than 2-fold increase in apoptosis of murine MDSCs was observed at 48 h with 500 nM PLX51107 (19% vs. 50%, *P* < 0.01), with both subsets being affected (Figure 4, C and D, and Supplemental Figure 7E). In vivo, a 1.4-fold increase in apoptosis was seen in splenic MDSCs from 4T1 tumor–bearing mice that received PLX51107 for 10 days (Figure 4E). Next, we determined if these findings could be replicated in experiments with human MDSCs. MDSCs isolated from the peripheral blood of patients with bladder cancer or melanoma were treated for 48 h with PLX51107, leading to a 1.5-fold increase of annexin V^+^ MDSCs (Figure 4, F and G; *P* < 0.0001 for melanoma, *P* < 0.01 for bladder cancer). The pan-caspase inhibitor Z-VAD-FMK blocked patient MDSC apoptosis, as measured by levels of cleaved caspase-3 (Figure 4, H and I; *P* < 0.0001). Of note, PLX51107 did not induce apoptosis in activated murine T cells (Supplemental Figure 7F). Lastly, we tested if BRD4 inhibitors could modulate suppressive molecules of MDSC, including *ARG1* and *NOS2*. At sublethal doses and time points, PLX51107 or JQ1 reduced *Arg1/ARG1* and *Nos2/NOS2* mRNA expression in MSC2 cells and murine and human MDSCs (Supplemental Figure 7, G–K). Together, these results implicate a role for BRD4 in the survival and suppressive functions of MDSCs.

### BRD4 regulates apoptotic proteins in MDSCs.

**Figure 5:**
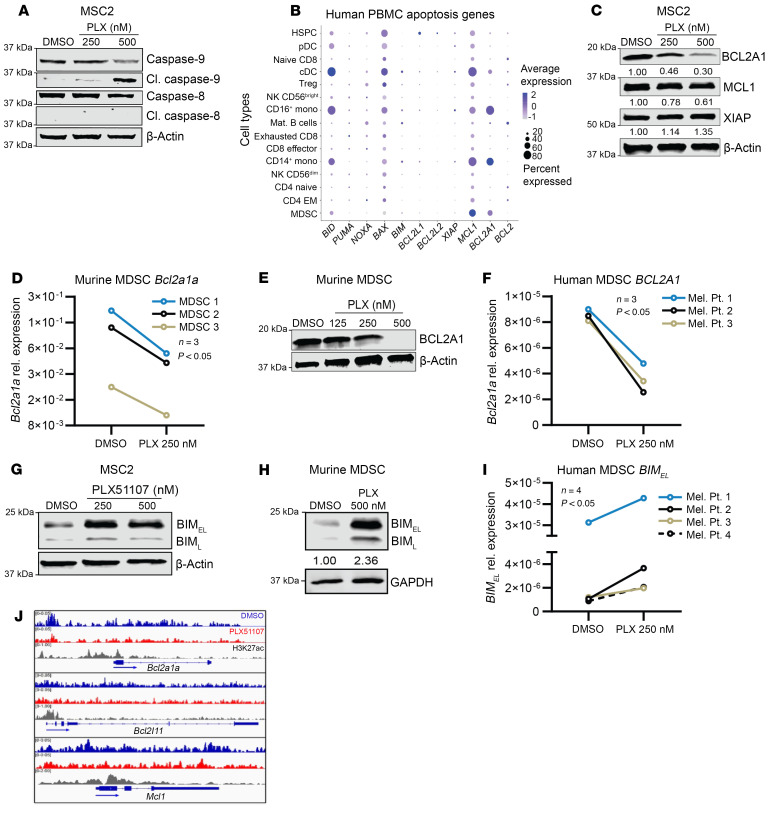
PLX51107 modulates apoptotic proteins in MDSCs. (**A**) MSC2 cells treated for 24 h with DMSO control or indicated dose of PLX51107 (PLX). Protein lysates of treated cells were probed with antibodies for caspase-9, cleaved caspase-9, caspase-8, cleaved caspase-8, and β-actin. (**B**) Single-cell RNA-Seq dataset of peripheral blood mononuclear cells of 16 patients at baseline. Dot plot of relative gene expression of select apoptosis genes across all cell types. (**C**) MSC2 cells treated as in **A** and probed for BCL2A1, MCL1, XIAP, and β-actin. (**D**) Splenic murine MDSCs were isolated from 4T1 tumor–bearing mice and treated for 48 h with DMSO control or the indicated dose of PLX51107 with media supplemented with 10 ng/mL IL-6 and GM-CSF. Relative gene expression of *Bcl2a1a* across treatment groups normalized to *β-actin*; *P* < 0.05 DMSO versus 250 nM PLX51107, paired 2-tailed Student’s *t* test. (**E**) Splenic murine MDSCs from 4T1 tumor–bearing mice treated as in **D**. Protein lysates from each condition probed with BCL2A1 or β-actin. (**F**) CD33^+^/CD11b^+^/HLA-DR^lo/–^ MDSCs were isolated from the peripheral blood of patients with melanoma by FACS. Cells were cultured in HAB media supplemented with 10 ng/mL IL-6 and GM-CSF and treated with DMSO or the indicated concentration of PLX51107. After 48 h, RNA was isolated from cells, and relative gene expression of *BCL2A1* across treatment groups was normalized to *18S*; *P* < 0.05 for DMSO versus 250 nM PLX51107, paired 2-tailed Student’s *t* test. (**G**) MSC2 cells treated as in **A** and probed for BIM. (**H**) Splenic murine MDSCs for 4T1 tumor–bearing mice treated as in **D** and probed for BIM. (**I**) Relative gene expression of *BIM_EL_* in human MDSCs treated as in **F** across treatment groups normalized to *18S*; *P* < 0.05 for DMSO versus 250 nM PLX51107, paired 2-tailed Student’s *t* test. (**J**) Gr1^+^/CD11b^+^ MDSCs were isolated from spleens of 4T1 tumor–bearing female mice by negative selection and treated with DMSO vehicle control (blue) or 125 nM PLX51107 (red) for 24 h. BRD4 enrichment was assayed by ChIP-Seq using input control. Control and inhibitor groups were normalized by *Drosophila* chromatin spike-in. Track heights indicate relative enrichment, and each group represents pooled cells from 3–4 mice. H3K27ac (gray) enrichment was determined from a public dataset (38).

To better understand the mechanism by which PLX51107 triggered MDSC apoptosis, MSC2 cells were treated with PLX51107 for 24 h, and protein lysates were probed for cleaved and full-length caspase-8 and caspase-9. PLX51107 appeared to induce apoptosis via the intrinsic pathway via caspase-9 cleavage, whereas caspase-8 cleavage was not detected (Figure 5A). To identify the genes that might be governing BRD4-mediated apoptosis in MDSCs, a single-cell RNA-Seq dataset of human PBMCs was analyzed. This dataset (ClinicalTrials.gov NCT03525925) included 16 patients with different malignancies with no prior checkpoint therapy. In the baseline samples, high expression of anti-apoptotic genes *MCL1* and *BCL2A1* in MDSCs from patients with cancer was observed compared with other cell types (Figure 5B). To determine which of these proteins might be regulated by BRD4 at the protein level, MSC2 cells were treated with PLX51107 for 24 h and examined by immunoblot. There was a 70% reduction in levels of BCL2A1 and a 39% decrease in levels of MCL1, but no decrease in XIAP (Figure 5C). Reduction of BCL2A1 was also seen in MSC2 cells that were treated with JQ1 (Supplemental Figure 7L). Additionally, PLX51107 treatment significantly reduced BCL2A1 mRNA and protein expression in murine MDSCs and *BCL2A1* mRNA expression in human MDSCs (Figure 5, D–F). To determine the direct role of BRD4 in regulating the transcription of *Bcl2a1a* and *Mcl1*, BRD4 ChIP-Seq was performed in splenic MDSCs from 4T1 tumor–bearing mice. BRD4 was detected in areas corresponding to H3K27ac enrichment determined from a dataset from de Almeida Nagata et al. (36), and treatment with PLX51107 decreased BRD4 occupancy (Figure 5J). Based on the work of Hu et al., which evaluated the role of BRD4 in the apoptosis of Eμ-Myc lymphoma, the levels of the pro-apoptotic protein BIM were analyzed (37). The induction of BIM protein was seen in MSC2 cells and murine MDSCs, while an increase of mRNA was observed in human MDSCs after PLX51107 treatment (Figure 5, G–I). Together, these results demonstrate that BRD4 inhibition induces apoptosis in MDSCs via modulation of specific proteins governing apoptosis, including a decrease in the anti-apoptotic protein BCL2A1 and an induction of the pro-apoptotic protein BIM.

### PLX51107 reduces cytokines implicated in MDSC expansion.

The fact that BRD4 inhibition could induce a small decrease in MDSCs within BRD4 myeloid–deficient mice with slightly smaller tumor volumes suggested the potential for the ability of inhibitors to exert a direct effect on tumor cells. BRD4 inhibition could theoretically prevent the release of tumor-derived soluble factors that are involved in the expansion and recruitment of MDSCs. To investigate this possibility, the Nanostring gene expression data from tumors treated with PLX51107 were further analyzed, and several soluble factors implicated in MDSC expansion were among the most downregulated genes. These included S100A8, IL-1β, IL-6, CSF3, and CSF2 (Table 1 in Supplemental Figure 8). To see if PLX51107 could reduce the expression of any of these factors, a Bio-Plex assay was run on conditioned media from 4T1 cells. Amongst these factors, only GM-CSF (62% decrease) and IL-6 (48% decrease) showed a significant reduction in vitro (Supplemental Figure 8A; *P* < 0.0001). PLX51107 treatment also resulted in a significant reduction of IL-6 in the plasma of EMT6 (95% reduction, *P* < 0.01) and 4T1 (78% reduction, *P* < 0.05) tumor–bearing mice (Supplemental Figure 8, B and C). GM-CSF was not detected in the plasma of either treatment group (data not shown). Tumor-secreted IL-6 and GM-CSF are known drivers of MDSC expansion, and the ability of PLX51107 to inhibit their production could contribute to the reduced frequency of MDSCs.

### BRD4 inhibitors block chemokine production by tumor cells, resulting in decreased MDSC migration.

Nanostring data from BRD4 inhibitor–treated tumors also revealed decreased tumor expression of chemokines important for MDSC recruitment to tumors including CXCL5, CXCL3, and CCL2, suggesting that the reduced intratumoral MDSC levels could be caused by impaired MDSC migration into the TME. CXCL5, CXCL3, and CCL2 protein expression by EMT6 and 4T1 cells was reduced by 55%–82% in response to in vitro PLX51107 treatment (Supplemental Figure 9, A–C and Supplemental Figure 9, D and F; *P* < 0.05 to *P* < 0.0001). Transwell migration assays were conducted using tumor-conditioned media (TCM) from DMSO or PLX51107-treated EMT6 and 4T1 cells as the stimulus for the migration of MDSCs. Use of TCM from PLX51107-treated cells led to 38%–50% less MDSC migration in EMT6 TCM, and this occurred in a dose-dependent fashion in 4T1 TCM (Supplemental Figure 9, G and H; *P* < 0.05 and *P* < 0.01, respectively). We next determined if these findings could be replicated with human MDSCs. Using TCM from the A375 melanoma cell line, PLX51107 treatment significantly reduced the migration of human MDSCs isolated from the peripheral blood of melanoma patients (Supplemental Figure 9I; *P* < 0.05). Similar to the mouse cell lines, PLX51107 treatment of human cancer cell lines resulted in reduced protein levels of CCL2 and CXCL5 (Supplemental Figure 9, J and K; *P* < 0.01). New interrogation of BRD4 ChIP-Seq data in the human TNBC cell line MDA-MB-231 published by Zanconato et al. (38) revealed BRD4 enrichment at the loci of MDSC-recruiting chemokines including IL-6, CXCL3, and CSF2 (Supplemental Figure 9L). Importantly, PLX51107 had no effect on tumor cell viability or levels of apoptosis at the 24-h time point when cells and TCM were collected for assays (Supplemental Figure 10, A and B), indicating that reduced chemokine expression was not the result of inhibitor-induced cell death. At an extended time point of 72 h, 4T1 and EMT6 tumor cell viability was still unaffected by PLX51107 up to doses of 1,000 nM (Supplemental Figure 10C).

### BRD4 inhibitors enhance T cell activation and the efficacy of checkpoint inhibitor therapy.

**Figure 6:**
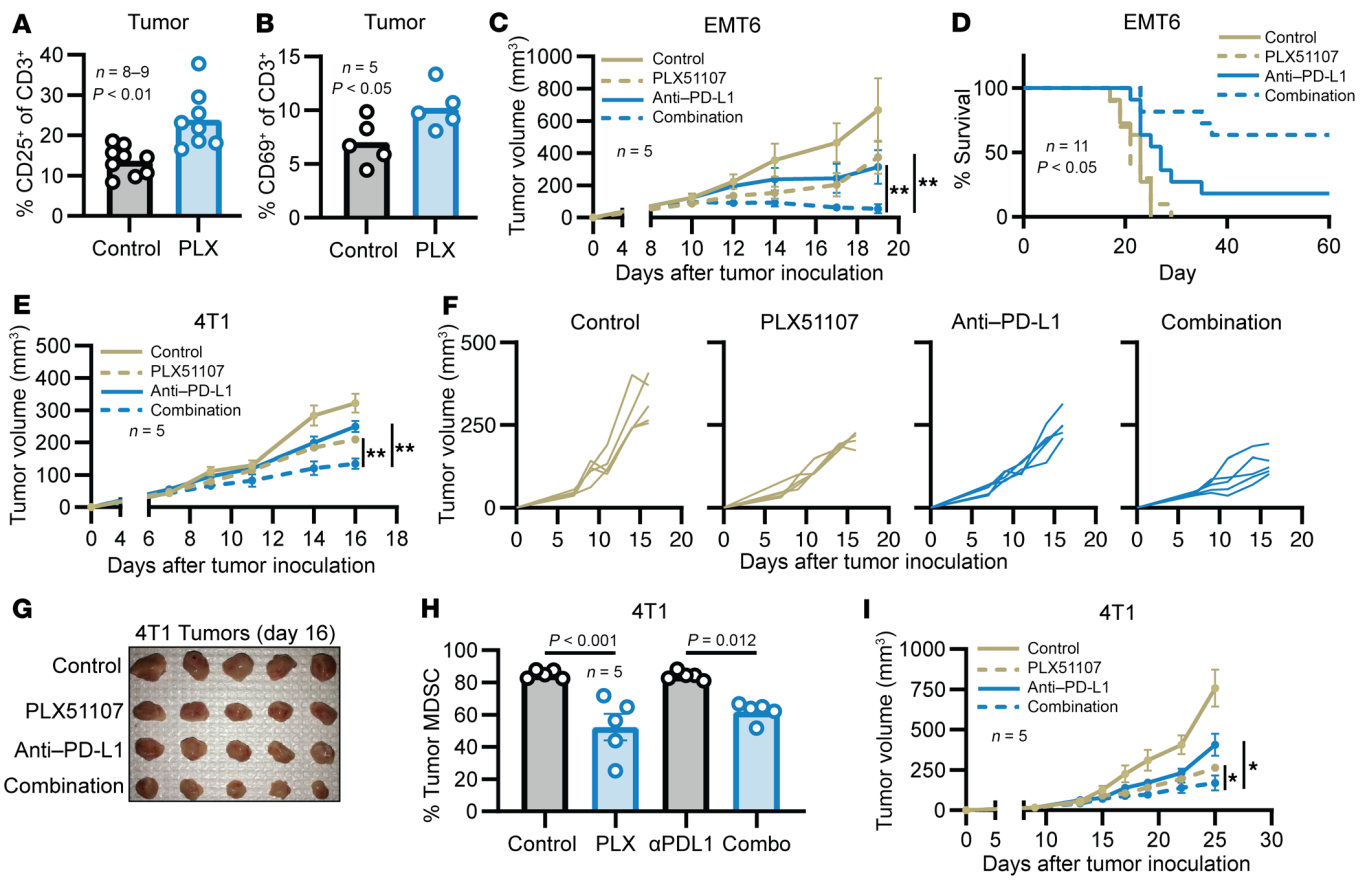
PLX51107 enhances the efficacy of anti–PD-L1 immune checkpoint therapy. (**A** and **B**) Frequency of intratumoral CD45^+^CD3^+^CD25^+^ (**A**) and CD45^+^CD3^+^CD69^+^ cells (**B**) analyzed by flow cytometry in EMT6 tumor–bearing mice treated for 1 week with control or PLX51107 (PLX). Values represent mean ± SEM; CD25: *P* < 0.01, *n* = 8–9 and CD69: *P* < 0.05, *n* = 5, unpaired 2-tailed Student’s *t* test. (**C**) EMT6 tumor–bearing mice were treated with control (vehicle and IgG), 20 mg/kg PLX51107 daily, 100 μg anti–PD-L1 3 times a week, or PLX51107 + anti–PD-L1. Tumor volumes were measured 3 times a week with digital calipers. Values are the mean ± SEM of tumor volumes at each time point (*n* = 5); *P* < 0.01 for combination versus PLX51107 and combination versus anti–PD-L1 (linear mixed model with Tukey-Kramer adjustment). (**D**) EMT6 tumor–bearing mice were treated as in **C**. Treatment was administered for 60 days or until institutional removal criteria were met, at which point treatment was stopped. Survival analyzed by 1-way ANOVA with Tukey’s correction; *P* < 0.05, *n* = 11. Long-term treatment did not elicit discernible toxicity. (**E**–**G**) The 4T1 tumor–bearing mice were treated as described in **A**. Values represent mean ± SEM of tumor volumes at each time point. Combination versus PLX51107 and combination versus anti–PD-L1; *P* < 0.01, linear mixed model with Tukey-Kramer adjustment. (**F**) Spider plot of tumor growth curves in **E**. (**G**) Images of 4T1 tumors at day 16. (**H**) 4T1 tumors were processed into single-cell suspensions. Values represent mean ± SEM of GR1^+^/CD11b^+^ MDSCs within the CD45^+^ population; *P* < 0.001 for control versus PLX51107, and *P* = 0.012 for anti–PD-L1 versus combination (Combo) treatment (1-way ANOVA with Tukey’s correction). (**I**) The 4T1 tumor–bearing mice were treated with control (vehicle and IgG), 20 mg/kg PLX51107 daily, 200 μg anti–LAG-3 3 times a week, or PLX51107 + anti–LAG-3. Tumor volumes were measured as in **C**. Values represent mean ± SEM of tumor volumes at each time point. Combination versus PLX51107: *P* < 0.05, *n* = 5; combination versus anti–LAG-3: *P* < 0.05, *n* = 5, linear mixed model with Tukey-Kramer adjustment, test results averaged over all days. *P < 0.05 and **P < 0.01.

To determine if reduced MDSC levels correlated with improved T cell function, T cell activation markers CD25 and CD69 were measured within EMT6 tumors. Intratumoral T cells from PLX51107-treated mice showed enhanced activation via increased frequency of CD25^+^ and CD69^+^CD3^+^ T cells (Figure 6, A and B; *P* < 0.01 for CD25 and *P* < 0.05 for CD69). There was a slight increase in the frequency of Tregs within the spleen of PLX51107-treated animals but no significant difference in the absolute number of Tregs (Supplemental Figure 11). Levels of exhausted CD8^+^ T cells were evaluated based on the work of Milner et al., who used a B16-GP_32_ model that demonstrated decreased exhaustion of tumor antigen–specific CD8^+^ T cells following BRD4 inhibition with JQ1 (39). In the 4T1 tumor model, there was a small but nonsignificant decrease in exhausted TIM3^+^/PD1^+^ double-positive intratumoral CD8^+^ T cells after PLX51107 treatment (Supplemental Figure 12, A and B).

Given the ability of BRD4 inhibitors to reduce the accumulation of MDSCs within tumors and the concurrent improvement of T cell activation, it was hypothesized that BRD4 inhibition might improve the efficacy of an immune checkpoint blockade. To test this hypothesis, mice bearing EMT6 tumors were treated with PLX51107 in combination with an anti–PD-L1 mAb. Control mice received the single agent or control solutions. Single-agent PLX51107 and anti–PD-L1 produced a moderate reduction in tumor volume, but the combination significantly reduced tumor growth compared with either agent alone (Figure 6C and Supplemental Figure 13A; *P* < 0.01). In addition, the combination produced a significant survival benefit in the EMT6 model compared with single-agent treatments (Figure 6D; *P* < 0.05). The survival benefit was driven by a high rate of complete tumor regression in the combination treatment group compared with single-agent anti–PD-L1 (Supplemental Figure 13B; 7/11 vs. 3/11 mice, *P* < 0.05). Regardless of treatment, all mice that experienced complete tumor regression were resistant to rechallenge with 1 × 10^6^ EMT6 cells. The ability of PLX51107 to enhance the efficacy of anti–PD-L1 therapy was also observed in the 4T1 model (Figure 6, E–G, and Supplemental Figure 13C; *P* < 0.01).

Analysis of tumor immune cell infiltrate demonstrated a significant 30% reduction of MDSCs within the EMT6 tumors treated with the combination of PLX51107 and anti–PD-L1 compared with anti–PD-L1 alone (Figure 6H and Supplemental Figure 14A; *P* < 0.05 or *P* < 0.01). Similarly, there was a reduction of total splenic MDSCs and MDSC subsets in the combination arm compared with anti–PD-L1 (Supplemental Figure 14, B–F). Thus, the effects of BRD4 inhibition on MDSC levels in spleens and tumors were equivalent whether given as a single agent or in combination with an anti–PD-L1 antibody. An analysis of T cells within the spleens of the 4T1 model showed a significant 50% increase in the frequency of CD4^+^ T cells with the administration of single-agent PLX51107 compared with the control. This effect on CD4^+^ T cells was also observed in mice receiving the combination treatment (Supplemental Figure 15A; *P* < 0.01). Further analysis revealed that the increase in CD4^+^ T cells was likely driven by an increase in the frequency of naive and effector memory CD4^+^ T cells (Supplemental Figure 15, B–E; *P* < 0.05 or *P* < 0.01). There was no substantial change in the frequency of CD8^+^ T cells or CD8^+^ T cell subsets (Supplemental Figure 16, A and E). To determine the ability of BRD4 inhibition to enhance the efficacy of other checkpoint inhibitors, PLX51107 was administered in combination with an anti–LAG-3 mAb in mice bearing 4T1 tumors. LAG-3–blocking Abs have shown efficacy in humans with advanced melanoma (40). As before, BRD4 inhibition significantly enhanced the antitumor effects of anti–LAG-3 therapy. Significantly smaller tumors were seen with the combination treatment compared with single-agent PLX51107 or anti–LAG-3 (Figure 6I; *P* < 0.05). Body weights of mice receiving the combination of PLX51107 and anti–LAG-3 were tracked, and body weight change did not warrant early removal (Supplemental Figure 16F).

**Figure 7:**
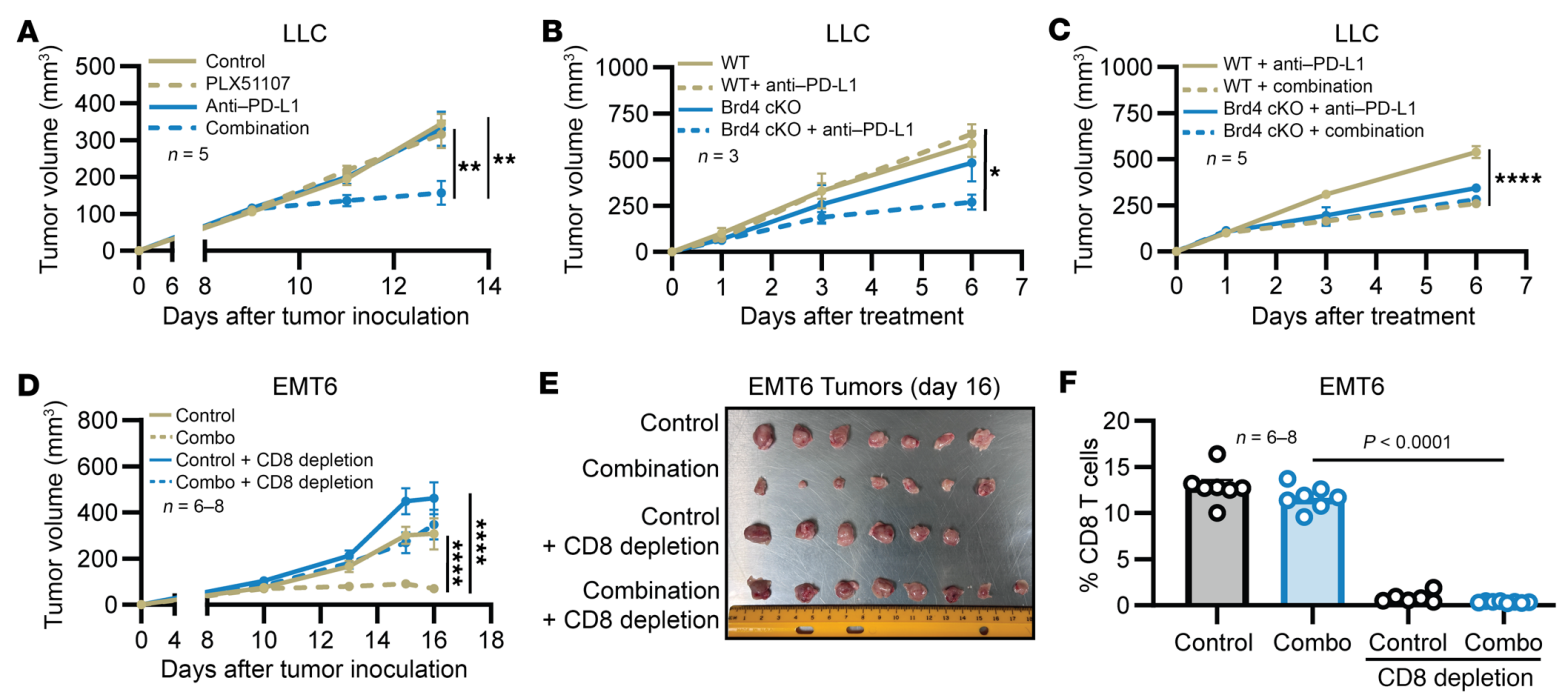
Immune checkpoint therapy enhancement by PLX51107 is dependent on myeloid BRD4 expression and CD8^+^ T cells. (**A**) LLC tumor–bearing mice were treated as described in Figure 6. Tumor volumes were measured 3 times a week with digital calipers. Values are the mean ± SEM of tumor volumes at each time point (*n* = 5); *P* < 0.01 for combination versus PLX51107 (PLX) and combination versus anti–PD-L1, linear mixed model with Tukey-Kramer adjustment, test results averaged over all days. (**B**) LLC tumor–bearing BRD4 WT or BRD4 cKO mice (100 mm^3^) received 100 μg of IgG or anti–PD-L1 on days 1, 3, and 6. Values represent mean ± SEM of tumor volumes at each time point, WT + anti-PDL1 versus cKO + anti–PD-L1 (*n* = 3); *P* < 0.05, linear mixed model with Tukey-Kramer adjustment). (**C**) LLC tumor–bearing BRD4 WT or BRD4 cKO mice (100 mm^3^) received 100 μg of IgG or anti–PD-L1 on days 1, 3, and 6. PLX51107 (20 mg/kg) or control was administered daily for 6 days. Values represent mean ± SEM of tumor volumes at each time point; WT + anti–PD-L1 versus WT + combination (*n* = 5), *P* < 0.000, linear mixed model with Tukey-Kramer adjustment. (**D**–**F**) EMT6 tumor–bearing mice received 200 μg of IgG or CD8 depletion antibody on day 7 and 100 μg of IgG or CD8 depletion antibody on days 10 and 14. On day 10, mice were treated with control (vehicle and IgG), 20 mg/kg PLX51107 daily, 100 μg anti-PDL1 Monday, Wednesday, and Friday, or a combination of PLX51107 and anti–PD-L1. Tumor volumes were measured 3 times weekly with digital calipers. (**D**) Values represent mean ± SEM of tumor volumes at each time point. Control versus combination (*n* = 7); *P* < 0.0001, combination versus combination + CD8^+^ depletion antibody (*n* = 6–8), *P* < 0.000, linear mixed model with Tukey-Kramer adjustment. (**E**) Tumor images at day 16. (**F**) CD3^+^CD8^+^ T cells were measured in the spleen by flow cytometry from Figure 7D. Values represent mean ± SEM of percent CD3^+^/CD8^+^ double-positive splenocytes. Combination versus combination + CD8^+^ depletion antibody (*n* = 6–8); *P* < 0.0001, 1-way ANOVA with Tukey’s correction. *P < 0.05, **P < 0.01, and ****P < 0.0001.

PLX51107 also enhanced anti–PD-L1 therapy in the LLC model, where the combination treatment again most strongly inhibited tumor growth paired with a reduction in total splenic MDSCs in the combination arm compared with anti–PD-L1 (Figure 7A and Supplemental Figure 17A; *P* < 0.01). To determine if the efficacy of anti–PD-L1 therapy in the LLC model was dependent on BRD4 expression in the myeloid compartment, BRD4 WT and BRD4 cKO mice bearing LLC tumors were treated with IgG control or anti–PD-L1 for 3 doses (days 1, 3, and 6). Anti–PD-L1 therapy did not reduce tumor volumes of BRD4 WT mice but was effective in mice deficient in BRD4 in the myeloid compartment (WT + anti–PD-L1 vs. cKO plus anti–PD-L1, *P* < 0.05) (Figure 7B). As previously seen, the administration of PLX51107 in combination with anti–PD-L1 in LLC tumor–bearing BRD4 WT mice led to a significant reduction in tumor volumes compared with anti–PD-L1 therapy alone (Figure 7, B and C; *P* < 0.0001). However, in the BRD4 cKO model, the administration of a BRD4 inhibitor did not improve the antitumor activity of anti–PD-L1 therapy (Figure 7C). These results suggest that the ability of BRD4 inhibition to improve the response to checkpoint inhibitor therapy is dependent upon the presence of this target within the myeloid compartment.

To evaluate if the effectiveness of PLX51107 in combination with anti–PD-L1 is dependent on CD8^+^ T cells, mice bearing EMT6 tumors were pretreated with a control antibody or an anti-CD8 antibody to deplete the CD8^+^ T cell population. Following depletion, EMT6 tumor–bearing mice were treated with a combination of PLX51107 and anti–PD-L1 or control reagents. The depletion of CD8^+^ T cells abrogated the efficacy of the combination therapy, suggesting its dependence on CD8^+^ T cells in the EMT6 model (Figure 7, D–F, and Supplemental Figure 17B). In fact, in the absence of CD8^+^ T cells, the combination therapy was no more effective than the control reagents. These results indicate the ability of the BRD4 inhibitor PLX51107 to enhance immune checkpoint therapy possibly by affecting the myeloid compartment and suggest its dependence on CD8^+^ T cells.

## Discussion

BRD4 inhibition has been shown to have a modest direct antitumor effect through induction of cell cycle arrest, apoptosis, and modulation of oncogene expression in cancer cells (1). However, as a single agent, these inhibitors have not mediated a strong anticancer effect. The present study provides evidence for an additional immunomodulatory role of BRD4 inhibitors, including direct pro-apoptotic effects on MDSCs and reduced tumor-soluble factors responsible for MDSC expansion and recruitment to the TME. We show that specific deficiency of BRD4 in the myeloid compartment led to significantly decreased levels of MDSCs in the setting of malignancy. Additionally, BRD4 inhibition enhanced the efficacy of both anti–PD-L1– and anti–LAG-3–blocking Abs. The reduced number of MDSCs in BRD4 cKO and mice receiving PLX51107 combined with its pro-apoptotic effects on MDSCs suggest a possible mechanism underlying the ability of BET inhibition to enhance the activity of checkpoint inhibitors, but this cannot be taken as definitive proof of a cause-and-effect relationship.

BRD4 inhibition likely reduced MDSC levels through multiple mechanisms. First, it was shown that BRD4 inhibition in MDSCs leads to apoptosis via caspase-9 intrinsic apoptosis pathway activation and modulation of apoptotic proteins, including anti-apoptotic BCL2A1 and pro-apoptotic BIM. Previous reports have found that caspase-9 intrinsic apoptosis pathways and expression of BCL2A1 play a role in overall MDSC survival (41, 42). Li et al. demonstrated the ability of 5-FU treatment to induce apoptosis of MSC2 cells and splenic MDSCs isolated from MCA205 tumor–bearing mice via caspase-9 activation (41). Medina-Echeverz et al. showed that the anti-apoptotic gene *Bcl2a1a* was upregulated in murine PMN-MDSCs by tumor-secreted GM-CSF, leading to their prolonged survival in the TME (42). In the present study, it was found that BRD4 inhibition via PLX51107 led to increased caspase-9 activation and significantly reduced BCL2A1 transcript and protein expression in MSC2, murine MDSCs, and human MDSCs. Additionally, it was found that BRD4 inhibition increased expression of BIM, which promotes apoptosis in both murine and human MDSCs. The suppression of BIM by BRD4 was also observed in vitro by Li et al. in a human hepatocellular carcinoma (HCC) cell line where BRD4 inhibition increased the expression of BIM, leading to HCC apoptosis (43). Together, we believe these results implicate BRD4 as a novel regulator for MDSC survival through modulation of apoptotic proteins.

PLX51107 is a BET inhibitor that also targets the bromodomains of BRD2, BRD3, and BRD4 and interacts with the bromodomains of CBP and EP300. However, the *K_D_* of interaction for those proteins is in the 100 nM range compared with <10 nM for BRD2, BRD3, and BRD4 (44). While BRD4 has been studied by us and others in the context of myeloid cells (2, 45–47), a potential role of BRD2 or BRD3 remains to be investigated. However, results from the BRD4 ChIP-Seq on MDSCs from tumor-bearing mice revealed an enrichment of BRD4 on key MDSC genes, highlighting the importance of BRD4 in MDSC biology.

BRD4 inhibition likely has effects on other immune compartments, but the importance of these actions is unclear. A study by Milner et al. found that BRD4 inhibition with the drug JQ1 led to decreased levels of the exhaustion markers TIM3^+^/PD1^+^ on intratumoral tumor antigen–specific CD8^+^ T cells that were adoptively transferred in the B16-GP_33_ model (39). In the present study, a small decrease of intratumoral TIM3^+^/PD1^+^ CD8^+^ T cells was observed in the 4T1 model after BRD4 inhibition. The discrepancy between the 2 studies may lie in the less-well-defined tumor antigen profile in the 4T1 model and the fact that antigen-specific CD8^+^ T cells could be specifically interrogated after being adoptively transferred to the B16-GP_33_ model. Notably, Milner et al. showed that BRD4 inhibition with JQ1 actually lowered the efficacy of immune-based therapies involving the adoptive transfer of tumor antigen–specific CD8^+^ T cells. Conversely, JQ1 treatment enhanced anti–PD-1 treatment in the MC-38GP_33_ model. However, the effect of BRD4 inhibition on the myeloid and T cell compartments was not studied. In the present study, enhancement of anti–PD-L1 therapy after BRD4 inhibition was seen in multiple tumor models, along with a significant reduction of both intratumoral and splenic MDSCs. Concurrently, the expression of activation markers CD69 and CD25 on intratumoral T cells was increased after BRD4 inhibition. Additionally, RNA-Seq was performed on tumors of 4T1 tumor–bearing mice treated with control or PLX51107, which was not initially reported (data not shown, our unpublished observations). An upregulation of LAG-3 was observed in response to PLX51107. This result suggested that the combination of PLX51107 and anti–LAG-3 may have efficacy in vivo, as demonstrated by the 4T1 model. Furthermore, LAG-3 upregulation in the TNBC TME has been associated with immunotherapy response (48), and TNBC patients have exhibited durable responses in a phase I/II study of combination anti–LAG-3 and anti–PD-1 therapy (49). Taken together, BRD4 inhibition mediates improved immune function in the setting of cancer via its inhibitory effects on MDSCs and the resulting release of T cell inhibition.

In the present study, BRD4 inhibitors significantly decreased IL-6 plasma levels in vivo and in vitro and led to a significant increase in the abundance of effector CD4^+^ T cells in lymphoid organs. This effect may well have been the result of a direct effect of BRD4 inhibition on growth factor secretion by tumor cells. BRD4 inhibitors also decreased tumor cell secretion of GM-CSF. The well-established role of IL-6 and GM-CSF in MDSC expansion and activation suggests that reduced MDSC growth factors following BRD4 inhibition may play a role in reducing MDSC numbers (50–52). Targeting IL-6 has also been shown to affect the TME and the response to anti–PD-L1 therapy. For example, in pancreatic cancer, IL-6 blockade resulted in increased effector T cell infiltration and enhanced efficacy of anti–PD-L1 therapy (53). PD-1/PD-L1 blockade itself resulted in increased IL-6 production by TAMs, resulting in a dysfunctional Th1 T cell response against melanoma (54). Depletion of macrophages or combined blockade of IL-6 and PD-1/PD-L1 restored Th1 T cell function and produced synergistic antitumor effects (54). Given the pleiotropic effects of BRD4 inhibition, it is possible that the significant decrease in IL-6 production contributed to the enhanced efficacy of BRD4 inhibitor and anti–PD-L1. It is also possible that inhibition of a tumor–MDSC interaction was interrupted after BRD4 inhibition, as additional tumor-secreted soluble factors, including chemokines (CCL2, CXCL3, and CXCL5), were all reduced after BRD4 inhibition in vitro. It is well established that these factors can recruit MDSCs into tumors (55).

BRD4 inhibition reduced the release of tumor-derived cytokines and chemokines, raising the possibility of an indirect effect on MDSCs via decreased generation of MDSCs and/or reduced intratumoral infiltration. To evaluate this mechanism in vivo, a genetic mouse model was employed (*Brd4^fl/fl^* *LysM-Cre*) in which mice were rendered deficient in BRD4 within the myeloid compartment. Loss of BRD4 in the myeloid compartment led to significantly decreased levels of MDSCs in tumor-bearing mice, suggesting an important role for BRD4 in MDSC expansion and survival. Notably, pharmacologic inhibition of BRD4 did not lead to a significant reduction of MDSCs within BRD4 myeloid–deficient mice. In the present study, it was found that single-agent anti–PD-L1 therapy had almost no antitumor effect in WT mice in the LLC model as tumor sizes were comparable to the control treatment. However, in BRD4 myeloid–deficient mice, single-agent anti–PD-L1 therapy resulted in reduced LLC tumor growth when compared with control treatment. Thus, specific loss of BRD4 in the myeloid compartment leading to reduced MDSC levels may be a possible mechanism for the increased efficacy of the combination therapy. The ability of BRD4 inhibition to increase the efficacy of anti–PD-L1 therapy in WT mice, but not in BRD4 myeloid–deficient mice, further supports the myeloid compartment as the critical target of PLX and JQ1. Overall, these results suggest that BRD4 is important in maintaining an immunosuppressive myeloid compartment.

While this study suggests that BRD4 inhibition acts predominantly by reducing MDSC abundance and recruitment to the tumor, other work has shown that BRD4 inhibition can alter immune macrophage phenotypes (15, 16). A study by Li et al. found that treatment of macrophages cultured in ovarian cancer cell line–conditioned media with the BRD4 inhibitor AZD5153 reversed their M2-like TAM phenotype with reduced expression of *ARG1*, *MRC1*, and *IL10* (15). Functionally, AZD5153-treated macrophages were less able to suppress CD8^+^ T cell activation. We saw no differences in intratumoral F4/80^+^ macrophages in mice receiving a BRD4 inhibitor. However, BRD4 inhibition decreased intratumoral M-MDSCs in multiple cancer models. The M-MDSC subset is known to transition into M2-like TAMs within the tumor, and the negative effect of PLX11507 on this population may play a role in shaping the intratumoral myeloid compartment in our studies (22). Additionally, BRD4 inhibition decreased *ARG1* and *NOS2* expression in human MDSCs at sublethal doses, confirming the possibility that reduced function or depletion of aberrant myeloid populations could be involved in the in vivo actions of BRD4 inhibition in combination with an immune checkpoint blockade.

As might be expected with a novel targeted agent, the combinatorial efficacy of BRD4 inhibitors and immune checkpoint inhibitors in solid tumors has been studied in the past and has been mostly attributed to direct tumor effects of BRD4 inhibition (56–60). This report presents a thorough immunologic mechanism behind the increased efficacy of the combination treatment, as it was found to be dependent on *BRD4* expression in the myeloid compartment and CD8^+^ T cells. A recent report by Aleckovic et al. explored the triple-combination therapy of BRD4 inhibitors in combination with anti–PD-L1 and paclitaxel in TNBC models. In both the EMT6 and MMTV-PyMT models, they noted increased infiltration of intratumoral T and B cells along with a shift in macrophage programming marked by MHCII^hi^ phenotype in the triple-combination treatment compared with control treatment (61). Interestingly, intratumoral CD11b^+^/Ly6G^+^ cells were found to be reduced in EMT6 tumor–bearing mice receiving JQ1. Although the authors did not speculate on the mechanism driving this reduction or whether these cells were PMN-MDSCs, our report suggests that decreased levels of CD11b^+^/Ly6G^+^ intratumoral cells can contribute to increased efficacy of their combination treatments.

This report demonstrates the ability of BRD4 inhibition to reduce MDSC levels and enhance the checkpoint blockade in multiple tumor models. A key mechanism leading to the loss of MDSCs was found to be through the direct apoptotic effect of BRD4 inhibitors on MDSCs. When paired with an immune checkpoint blockade in vivo, BRD4 inhibitors significantly improved the efficacy of anti–PD-L1 and anti–LAG-3 therapy, resulting in overall reduced tumor growth and improved survival. This enhancement of immune checkpoint blockade efficacy was found to be dependent on BRD4 expression in the myeloid compartment as well as CD8^+^ T cell activity. Thus, BRD4-induced maintenance of MDSC immune suppression could serve as a druggable target in the context of immune-based therapies.

## Methods

### Sex as a biological variable.

For murine breast cancer models, our study exclusively examined female mice because the disease modeled is primarily relevant in females. For other cancer models, both sexes were used.

### Cell lines.

MSC2 (murine MDSC cell line) and tumor cell lines (EMT6, 4T1, C26, LLC, A375, and MIA PaCa-2) were used as described. Detailed descriptions can be found in the Supplemental Methods.

### The Cancer Genome Atlas analysis.

TIMER2.0 was used to analyze the relationship between *BRD4* and MDSC gene signature in breast cancer patients based on stage (http://timer.cistrome.org) (29). The TIDE tool was used to analyze the relationship between MDSCs and TAM infiltration across a pan-cancer database (http://tide.dfci.harvard.edu) (28).

### Murine tumor models.

Female 4- to 6-week-old BALB/c mice (The Jackson Laboratory) were injected with respective cell lines and treated as described. Detailed descriptions can be found in the Supplemental Methods.

### NSG-SGM3 murine model.

Immunodeficient NOD.Cg-*Prkdc^scid^ Il2rg^tm1Wjl^* Tg (CMV-IL-3, CSF2, KITLG)1Eav/MloySzJ (NSG-SGM3; stock 013062) mice were engrafted with umbilical cord blood–derived HSCs. Female CD34^+^ HSC-engrafted NSG-SGM3 mice co-engrafted with the PDX tumor model TM01149 were used and treated as described. Detailed descriptions can be found in the Supplemental Methods.

### C57BL/6 BRD4^fl/fl^ LysM-Cre mice.

C57BL/6 *BRD4^fl/fl^ LysM-Cre* mice were used to delineate the effect of loss of BRD4 in the myeloid compartment. Detailed descriptions can be found in the Supplemental Methods.

### Isolation of murine MDSCs from tumor-bearing mice.

Spleens were harvested aseptically from mice, and CD11b^+^/GR1^+^ MDSCs were isolated using a murine MDSC isolation kit (STEMCELL Technologies) with purity > 95% by flow cytometry (21). MDSC subsets were isolated using an MDSC isolation kit (Miltenyi Biotec) with purity > 95% by flow cytometry (20). Detailed reagent specifications can be found in Supplemental Table 1.

### Isolation of human MDSCs and T cells from patients with cancer.

Peripheral human MDSCs and T cells were isolated from patients with stage 4 melanoma or bladder cancer via FACS based on expression markers and used as described. Detailed descriptions can be found in the Supplemental Methods.

### Flow cytometry analysis.

Spleens and tumors from mice were processed into single-cell suspensions and stained with the fluorochrome-labeled antibodies listed in Supplemental Tables 1 and 2. A detailed description can be found in the Supplemental Methods.

### IHC.

IHC (GR1, F480, and CD206) was performed by the Comparative Pathology and Digital Imaging Shared Resource at The Ohio State University and Histowiz. Detailed descriptions can be found in the Supplemental Methods and in Supplemental Table 1.

### Nanostring analysis.

Isolated mRNA was used for digital gene expression analysis of 730 immune genes and key inflammatory pathways, using the nCounter PanCancer Immune Profiling Panel (NanoString Technologies) on the nCounter Gene Expression Assay (NanoString Technologies) as previously described (62). In brief, 100 ng of mRNA was hybridized with sequence-specific barcoded probes at 65°C for 24 h before being placed into the NanoString Prep Station where the target-probe complex was immobilized onto the analysis cartridge. Cartridges were read by the nCounter Digital Analyzer for digital counting of molecular barcodes corresponding to each target.

### Real-time PCR.

MSC2 and murine or human MDSCs were treated in media containing DMSO (control) or PLX51107 for 24 or 48 h. After treatment, cells were harvested with trypsin, pelleted, and processed into RNA with the mirVana isolation kit (Thermo Fisher Scientific). The RNA concentration and purity were validated using a NanoDrop spectrophotometer (Thermo Fisher Scientific), and total RNA was reverse transcribed. cDNA was used to quantify gene expression using SYBR green and/or TaqMan (Thermo Fisher Scientific) with the following primers: *Bcl2a1a* (murine), *β-actin* (murine), *BCL2A1* (human), *β-actin* (human), *BCL2L11* (*BIM*) (human), *ARG1* (murine and human), *NOS2* (murine and human), and 18S (Integrated DNA Technologies). Detailed reagent specifications can be found in Supplemental Table 1.

### Immunoblot.

The murine MDSC cell line MSC2 or splenic murine MDSCs freshly isolated from tumor-bearing mice were treated for 24 or 48 h with DMSO (control), PLX51107, or JQ1. Cells were lysed in RIPA buffer (Sigma) with protease and phosphatase inhibitors. Lysates were probed on nitrocellulose membranes with antibodies as described. Detailed information can be found in the Supplemental Methods and Supplemental Table 1.

### scRNA-Seq dataset analysis.

Data from ClinicalTrials.gov (NCT03525925) were used. The study was conducted at The Ohio State University Comprehensive Cancer Center under an IRB-approved protocol (IRB protocol 2018C0070). Expression of each apoptotic gene was calculated based on the relative expression of each gene across all cell types and plotted using the DotPlot function in Seurat. Detailed descriptions can be found in Supplemental Methods.

### BRD4 ChIP-Seq in murine MDSCs.

Splenic CD11b^+^/Gr1^+^ MDSCs were isolated from spleens of female 4T1 TNBC tumor–bearing BALB/c mice and treated for 24 h with DMSO vehicle control or 125 nM PLX51107. Cells were fixed, and BRD4 ChIP-Seq was performed by Active Motif. Detailed descriptions can be found in Supplemental Methods.

### Statistics.

Statistical differences between more than 2 treatment groups were determined using a 1-way ANOVA model with Tukey’s correction. Two-group comparisons were performed using unpaired 2-tailed Student’s *t* test unless otherwise noted. One-way ANOVA model with Tukey’s correction and Student’s *t* tests were conducted in GraphPad Prism v10. For murine tumor studies, tumor volume data were log transformed first, and linear mixed model and contrasts were used for comparing different treatment groups. Tukey’s method was used for multiplicity adjustment comparisons done at each time point and averaged across all time points using *t* statistics by a statistician.

### Study approval.

Murine tumor model studies were conducted under a protocol approved by The Ohio State University’s IACUC (2009A0179-R3). The NSG-SGM3 murine study was conducted by In Vivo Services at The Jackson Laboratory’s Sacramento facility, an Office of Laboratory Animal Welfare–assured and American Association for Accreditation of Laboratory Animal Care–accredited organization, and was performed according to an IACUC-approved protocol and in compliance with the *Guide for the Care and Use of Laboratory Animals* (National Research Council, 2011). Patient PBMC samples were procured following patient signing of written informed consent conducted in accordance with recognized ethical guidelines under IRB-approved protocols for human subject research (IRB protocols 199C0348, 2004C0096, 2010C0036 and 2018C0070).

### Data availability.

All reported data values are available in the Supporting Data Values file. Supporting analytic code is available upon reasonable academic request to the corresponding author. Raw and processed ChIP-Seq files are available in Gene Expression Omnibus under accession number GSE302852.

## Author contributions

WEC conceived the project. HS, AS, AL, RW, and MCD designed the experiments. RW, RCW, DS, MH, and KLK provided patient samples. HS, AS, AL, ES, ICG, MCD, SZ, GL, AA, L Savardekar, L Scarberry, RP, JS, BB, and SHS performed the experiments and analyzed the data. HS, AS, AL, ICG, MCD, MJD, DQ, JL, LY, and AD interpreted the data. HS, AS, and AL wrote the manuscript. HS, AS, AL, DQ, GX, AD, KO, and WEC reviewed and edited the manuscript. The order of the co–first authors was assigned based on their specific contributions and efforts to the study.

## Supplementary Material

Supplemental data

Unedited blot and gel images

Supporting data values

## Version 1 08/05/2025

In-Press Preview

## Version 2 10/01/2025

Electronic publication
