# Supplementary material for: BRD4 inhibition leads to MDSC apoptosis and enhances checkpoint blockade therapy
Source: J Clin Invest. 2025 Aug 5;135(19):e181975. doi: 10.1172/JCI181975 (PMC12483567; doi:10.1172/JCI181975)
Supplement: Supplemental data [file jci-135-181975-s063.pdf]

Supplemental Methods

Cell lines

The murine MDSC cell line MSC2 (gift from Dr. Gregoire Mignot) was cultured in RPMI 1640 media supplemented with 25 mM HEPES, 10% heat-inactivated fetal bovine serum (FBS), 1% antibiotic-antimycotic, and 1 mM sodium pyruvate (52). Cell lines (EMT6, 4T1, C26, LLC, A375, MIA PaCa-2) lines were purchased from ATCC. EMT6, 4T1, and C26 were maintained in complete RPMI media. LLC, A375, and MIA PaCa-2 were maintained in complete DMEM media. All cell lines were acquired after 2010 and were validated by karyotyping/cytogenetic analysis.

Murine tumor models

Female 4–6 week old BALB/c mice (Jackson Laboratories, Bar Harbor, ME) were injected with 10^6 EMT6, 10^6 C26, or 10^5 4T1 cells in the mammary fat pad to produce tumors. 4–6 week old C57BL/6 mice (Jackson Laboratories, Bar Harbor, ME) were injected subcutaneously with 10^6 LLC cells. PLX51107 (Plexxikon) 20 mg/kg, JQ1 (MedChemExpress) 50 mg/kg, PLX2853 (Plexxikon) 5 mg/kg or vehicle (NMethyl pyrrolidone (NMP)) was administered by oral gavage daily (53). Anti-PDL1 (BioXcell cat#BE0101) and anti-LAG3 (BioXcell cat#BE0174) monoclonal antibodies (mAb) were administered by intraperitoneal injection at 100 µg thrice weekly (21, 54). Anti-CD8a mAb (BioXcell cat#BE0061) was administered by intraperitoneal injection at an initial dose of 200 µg followed by weekly 100 µg doses. Appropriate IgG controls were used (Bio X cell).

NSG-SGM3 murine model

Immunodeficient NOD.Cg-*Prkdc*^{scid} *Il2rg*^{tm1Wjl} Tg (CMV-IL3, CSF2, KITLG)1Eav/MloySzJ (NSG-SGM3, Stock# 013062) were engrafted with umbilical cord blood-derived hematopoietic stem cell (HSC). Female $CD34^+$ HSC-engrafted NSG-SGM3 mice co-engrafted with the PDX tumor model TM01149 were used. Two $CD34^+$ HSC donors were used. Mice were distributed across treatment groups such that tumor volume and variance was approximately equal. Tumor volume

at enrollment was 60-200 mm^3. Mice were also distributed evenly across groups by human leukocyte engraftment (% $hCD45^+$ cells). Mice were at least 12-weeks post $CD34^+$ HSC engraftment at the time of enrollment. Mice were treated daily with vehicle or PLX51107 (20mg/kg). This study was conducted by In Vivo Services at The Jackson Laboratory Sacramento facility, an OLAW-assured and AAALAC-accredited organization (30). This study was performed according to an IACUC-approved protocol and in compliance with the Guide for the Care and Use of Laboratory Animals (National Research Council, 2011).

C57BL/6 $BRD4^{fl/fl}$ LysMcre mice

C57BL/6 *$BRD4^{fl/fl}$ LysMcre* mice were genotyped using 2% agarose gel electrophoresis using BRD4 forward and reverse primers. Presence of an 1.1 kb BRD4 floxed allele and the absence of the 1.0 kb BRD4 WT allele indicated *$BRD4^{fl/fl}$* mice as previously described (12). LysMcre expression was verified by the presence of a 700 kb band using a 2% agarose gel (Jackson Lab protocol: 26499 as previously described (12)). Loss of BRD4 expression in the murine myeloid compartment was verified by isolating splenic murine MDSC from tumor-bearing mice and probing for BRD4 via immunoblot (Bethyl Labs). Bactin was used as a loading control. Immunoblot procedures were conducted as described below. Detailed reagent specifications can be found in **Supplemental Table 1.**

Isolation of human MDSC and T cells from patients with cancer

Whole blood was collected from patients with stage IV melanoma patients or bladder cancer. Approximately 30 mL of peripheral venous blood was collected from each patient. Peripheral blood mononuclear cells (PBMC) were isolated from peripheral venous blood via density gradient centrifugation with Ficoll, as previously described (55). Briefly, samples were layered carefully on top of the Ficoll density gradient medium (density = 1.077 g/mL) and centrifuged for 25 minutes at 1500 rpm with the brake off. Using this isolation method, PMN-MDSC remain in the PBMC

fraction while PMN are pelleted out over Ficoll. Enriched cell populations were removed from the density gradient medium (plasma interface) and residual red blood cells were lysed. PBMC underwent fluorescence activated cell sorting (FACS) and MDSC were isolated based on $CD11b^{+}$/ $CD33^{+}$/HLA-DR^{low} expression (75-80% purity) and T cells were isolated based on $CD3^{+}$ expression (56). MDSC and T cells were used immediately in the experiments described below.

Generation of tumor-conditioned media

To generate tumor-conditioned media (TCM), approximately 2×10^{5} cancer cells (EMT6, 4T1, A375, MIA PaCa-2) per 1 mL were grown to 80% confluence and incubated in 0.2% FBS medium with 0.2% DMSO or PLX51107 for 24 hours (21). After incubation, the TCM was harvested and centrifuged to remove suspended cells.

Migration assays

Murine MDSC of MDSC from metastatic melanoma patients were plated in the top chamber of an 8 μm Transwell assay. Tumor-conditioned media was used to stimulate MDSC migration. Cells in the bottom well were counted via CountBright beads (Thermo-Fisher) to determine the number of cells migrated in response to TCM as described (21).

Cytokine/chemokine measurement

Tumor cell lines were treated with DMSO or PLX51107 for 24h. Supernatants were collected after 24 hours, and levels of cytokines and chemokines were measured using enzyme-linked immunosorbent assays (BDBioscience) or Luminex multiplex assays.

Flow cytometric analysis

Spleens and tumors from mice were processed into single-cell suspensions and stained with fluorochrome-labeled antibodies found in **Supplemental Table 1**. Cells were analyzed using a

Fortessa flow cytometer (BD Biosciences). For apoptosis and proliferation measurements, human and murine MDSCs were cultured with PLX51107, JQ1 or 0.2% DMSO with 10 ng/mL IL-6 and GM-CSF (Peprotech) for 24 and 48h. Cells were harvested, stained with Annexin V/PI (BD Bioscience), cleaved caspase 3 fluorochrome-labeled antibody (BDBioscience) or Ki67 antibody (Biolegend) and analyzed using a Fortessa flow cytometer (BDBiosciences).

For spectral flow cytometry, samples were stained with BD Fixability Viability Stain 440UV (Becton Dickinson) followed by blocking with TrueStain FcX Block (Biolegend) and normal mouse sera (Invitrogen) for 15 minutes on ice. After blocking, samples were stained with extracellular surface markers for 30 minutes at 4C (**Supplemental Table 2**). Samples then underwent fixation and permeabilization with Foxp3/Transcription Factor Staining Buffer Set per manufactures instructions (eBioscience). All samples were acquired on Cytek Aurora 5L (Cytek Biosciences) and results were analyzed with OMIQ Flow Cytometry software (Dotmatics) and GraphPad Prism (Dotmatics).

Immunohistochemistry

Immunohistochemistry for F4/80, GR1, and CD206 was performed by the Comparative Pathology and Digital Imaging Shared Resource at the Ohio State University and Histowiz. Briefly, formalin-fixed, paraffin-embedded EMT6 breast cancer tumors were sectioned at 5 μM and mounted on Superfrost Plus slides. Following deparaffinization and antigen retrieval slides were incubated for 30 minutes with primary antibody anti-F4/80 (1:200, #MCA497G, Bio-Rad (formerly Serotec)) or anti-GR1 (1:100, #557445, BD Pharmingen) followed by incubation with secondary antibody for 30 minutes (rabbit anti-rat, 1: 200, BA-4000-1.5, Vector Laboratories). Detection of immune complexes was performed by using Vector ABC HRP RTU Elite (avidin–biotinylated enzyme complex, Vector Laboratories) with 3,3′-diaminobenzidine (DAB) as the chromogenic substrate, according to the manufacturer's recommendations, then counterstained with

Hematoxylin. Positive labeling was interpreted and quantified by a pathologist (MJD) (22). Quantification of F4/80 and GR1 tissue immunoreactivity was determined by the mean count of positive cells in five 400x magnification fields. Detailed reagent specifications can be found in **Supplemental table 1.**

Nanostring analysis

Isolated mRNA was analyzed for digital gene expression analysis of 730 immune genes and key inflammatory pathways, using the nCounter® PanCancer Immune Profiling panel (NanoString Technologies, WA, USA) on the nCounter® Gene Expression Assay (NanoString Technologies) as previously described (57). In brief, 100 ng of mRNA was hybridized with sequence-specific barcoded probes at 65°C for 24 h before being placed into the Nanostring Prep Station where the target-probe complex was immobilized on to the analysis cartridge. Cartridges were read by the nCounter Digital Analyzer for digital counting of molecular barcodes corresponding to each target.

Immunoblot

The murine MDSC cell line MSC2 or splenic murine MDSC freshly isolated from tumor-bearing mice were treated for 24 or 48h with DMSO (control), PLX51107, or JQ1. Cells were lysed in RIPA buffer (Sigma) with protease and phosphatase inhibitors. Lysates were probed on nitrocellulose membranes for caspase 8, cleaved caspase 8, caspase 9, cleaved caspase 9, XIAP, MCL1, BCL2A1, BIM, β-actin, or GAPDH. Membranes were incubated overnight in primary antibody dissolved in Blocker BLOTTO in TBS (Thermo-Fisher). After a 1 h room temperature incubation with species-appropriate secondary antibody, membranes were imaged on a LI-COR Odyssey CLx device (Lincoln, NE). Detailed reagent specifications can be found in **Supplemental Table 1.**

Analysis of scRNA-seq dataset

Data from ClinicalTrials.gov ID # NCT03525925 was used. The study was conducted at the Ohio State University Comprehensive Cancer Center under an Institutional Review Board (IRB)-approved protocol (IRB protocol no. 2018C0070). All patients were required to understand and sign an IRBapproved informed consent prior to screening and study-related procedures. Raw sequencing data of PBMC from 16 patients with different malignancies at the baseline timepoint was downloaded from GEO (accession number GSE178882). Gene expression counts were normalized, log-transformed, and scaled by 'SCTransform' in R software package Seurat (4.0.0) (58). Principal components were calculated from the top 2000 variable genes and the top 20 principal components (PCA) were selected to generate Uniform Manifold Approximation and Projection (UMAP) (59). Clusters of similar cells were detected using the Louvain algorithm and UMAP coordinates were used to construct a shared nearest neighbor graph by 'FindNeighbors' function at 0.5 resolution (60). Using FindAllMarkers function in the Seurat to assign cell-type signatures, logfc.threshold =0.25 (61) and adjusted by cellular detection rate. Dead cells and doublets were removed by the high concentration of mitochondrial genes (62). Blueprint (63), Encode (64), and Hao Y's single-cell PBMC data (65) were leveraged to annotate single cell types using the SingleR (66) package. Expression of each apoptotic gene was calculated based on the relative expression of each gene across all cell types and plotted using function 'DotPlot' from Seurat.

Supplemental Table 1: Reagent Information

Flow cytometry Antibody	Species	Supplier	Catalog Number	Fluorophore
CD11b	Human	Biolegend	301306	PE
CD33	Human	Biolegend	366606	APC
HLA-DR	Human	Biolegend	307616	PE-Cy7
CD11b	Human	Biolegend	101206	FITC
CD45	Mouse	Biolegend	103108	FITC
CD11b	Mouse	Biolegend	101208	PE
GR1	Mouse	Biolegend	108433	Pacific Blue
Ly6C	Mouse	Biolegend	128032	Pacific Blue
Ly6G	Mouse	Biolegend	127614	APC
Annexin V		BD Biosciences	550474	APC
Annexin V		BD Biosciences	51-65874X	FITC
Propidium Iodide		BD Biosciences	51-66211E	PE
Cleaved Caspase 3 Staining kit		BD Biosciences	550914	PE
Ki67		Biolegend	652403	PE
CD45	Mouse	Biolegend	103129	Per-CP-A
FOXP3 Staining kit	Mouse	eBioscience	00-5523-00	APC
CD3	Mouse	Biolegend	100220	PE-Cy7
CD69	Mouse	Biolegend	104506	FITC
CD25	Mouse	Biolegend	102007	PE
CD8	Mouse	Biolegend	100712	APC
TIM3	Mouse	Biolegend	134003	PE
CD8	Mouse	Biolegend	100722	PE-Cy7
CD4	Mouse	Biolegend	1004321	PerCP
CD44	Mouse	Biolegend	103012	APC
CD62L	Mouse	Biolegend	104408	PE
CD3	Mouse	Biolegend	100214	Pacific Blue

Immunoblot Antibody	Species	Supplier	Catalog Number	Dilution
Caspase 8	Rabbit	Cell signalling	47903	1:1000
Cleaved Caspase 8	Rabbit	Cell signalling	8592T	1:1000
Caspase 9	Rabbit	Cell signalling	9504T	1:1000
Cleaved Caspase 9	Rabbit	Cell signalling	9509S	1:1000
XIAP	Rabbit	BD Biosciences	610762	1:1000
MCL1	Rabbit	Proteintech	16225-1-AP	1:1000
BCL2A1	Rabbit	Cell signalling	64310S	1:1000
BIM	Rabbit	Cell signalling	29335	1:1000
B-Actin	Mouse	Proteintech	60008-1	1:2000
GAPDH	Mouse	Proteintech	60004-1	1:2000
BRD4	Rabbit	Bethyl Labs	A301-985A50	1:1000

Immunoprecipitation Antibody	Species	Supplier	Catalog Number
BRD4	Rabbit	Active Motif	91301

PCR Primer	Species	Supplier	Assay ID/ Catalog number
BCL2A1	Human	Integrated DNA Technologies	Hs.PT.56a.1995943
B-actin	Human	Integrated DNA Technologies	Hs.PT.39a.22214847
Bcl2a1a	Mouse	Integrated DNA Technologies	Mm.PT.58.33467370.g
B-actin	Mouse	Integrated DNA Technologies	Mm.PT.39a.22214843.g
18s	Eukaryotic	ThermoFisher	4310893E
BIM	Human	Integrated DNA Technologies	Hs.PT.58.18885697
Arg1	Mouse	Integrated DNA Technologies	Mm.PT.58.8651372
ARG1	Human	Integrated DNA Technologies	Hs.PT.56a.20779559
Nos2	Mouse	Integrated DNA Technologies	Mm.PT.58.43705194
NOS2	Human	Integrated DNA Technologies	Hs.PT.58.14740388

Genotyping Primers

Brd4-9618F: CCTGTGTGCACTTGCTCCCGAGGAGAGA

Brd4-9621R: GGACTAGAAACCTCCCAAATGTCTACAA

Supplemental Table 2: Spectral Flow Cytometry Panel

Flow cytometry Antibody	Clone	Supplier	Catalog Number	Fluorophore
CD206	Y14-505	BD Biosciences	568817	BUV395
CD11c	HL3	BD Biosciences	751265	BUV615
CD19	1D3	BD Biosciences	612781	BUV737
CD45	30-F11	BD Biosciences	568336	BUV805
Ly6C	HK1.4	Biolegend	128014	Pacific Blue
CD4	RM4-5	BD Biosciences	563106	BV510
CD14	Sa2-8	Invitrogen	63-0141-82	SuperBright 600
Ly6G	1AB	BD Biosciences	563979	BV711
CD3	17A2	Biolegend	100231	BV785
CD49b	HMa2	BD Biosciences	755482	RB780
F4/80	T45-2342	BD Biosciences	752152	R718
CD8	53-6.7	BD Biosciences	557654	APC-Cy7
CD11b	M1/70	BD Biosciences	566416	BB700
Fixable Viability Stain		BD Biosciences	566332	440 UV

Figure S1

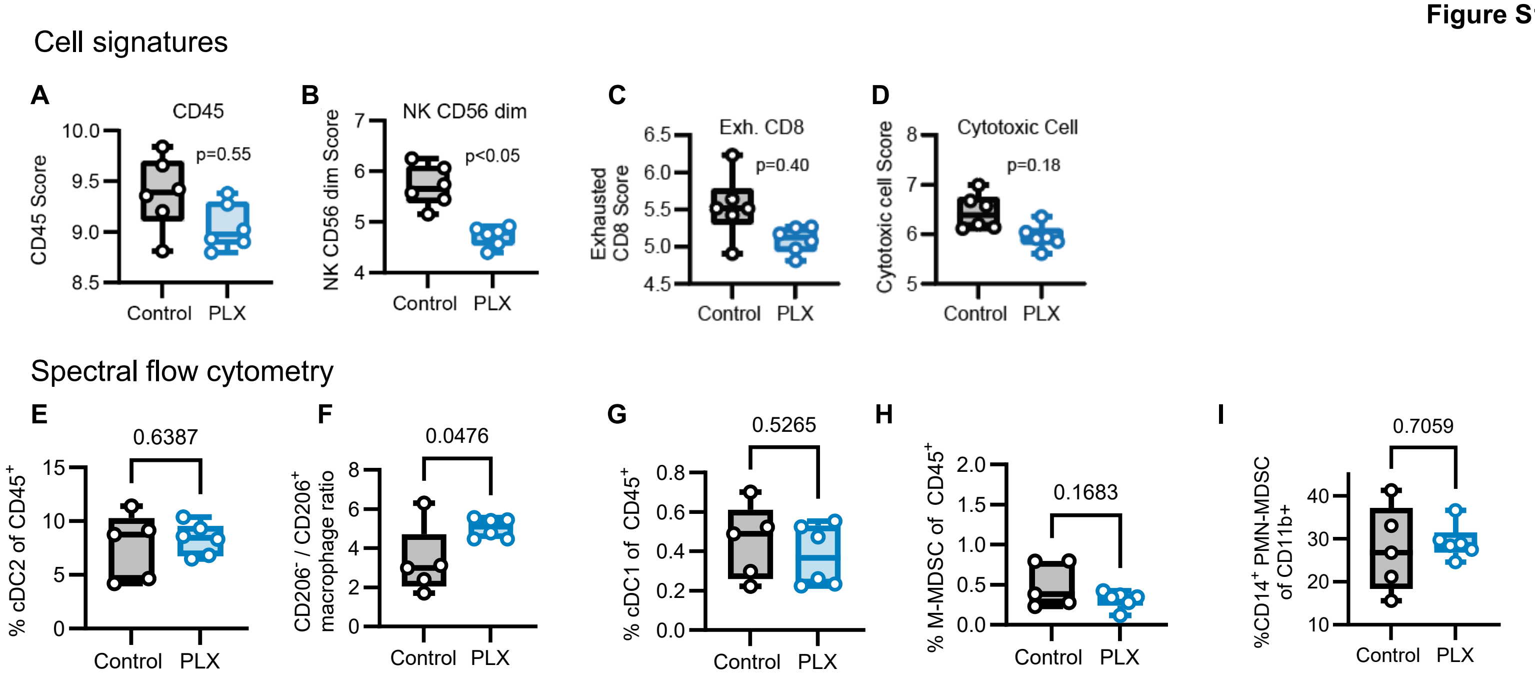

Figure S1. PLX51107 alters tumor composition

(A-D). BALB/c mice were inoculated with 1x10^6 EMT6 cells and treated with vehicle (control) or 20 mg/kg PLX51107 daily p.o once tumors were palpable (50 mm^3). After one week of treatment, RNA was extracted from whole tumors. Gene expression was then analyzed using the Nanostring PanCancer immune profiling panel. Raw cell abundance scores for indicated cell types are displayed on a logarithmic scale. A difference in the mean raw abundance score of 1 between control and PLX51107 treatments indicates a two-fold difference. ANOVA models and t-statistics were used for the comparison of cell type scores (log2) between control and PLX51107.

(E-I) BALB/c mice were inoculated with 1x10^5 4T1 cells and treated with vehicle (control) or 20 mg/kg PLX51107 daily p.o once tumors were palpable (50 mm^3). After 8 days of treatment, tumors were processed into single-cell suspensions and stained with fluorescent antibodies. Cell populations were acquired by spectral flow cytometry and unmixed on the Cytek Aurora 5L cytometer and processed using the OMIQ software platform (see Supplemental Table 2 for full antibody panel).

Figure S2

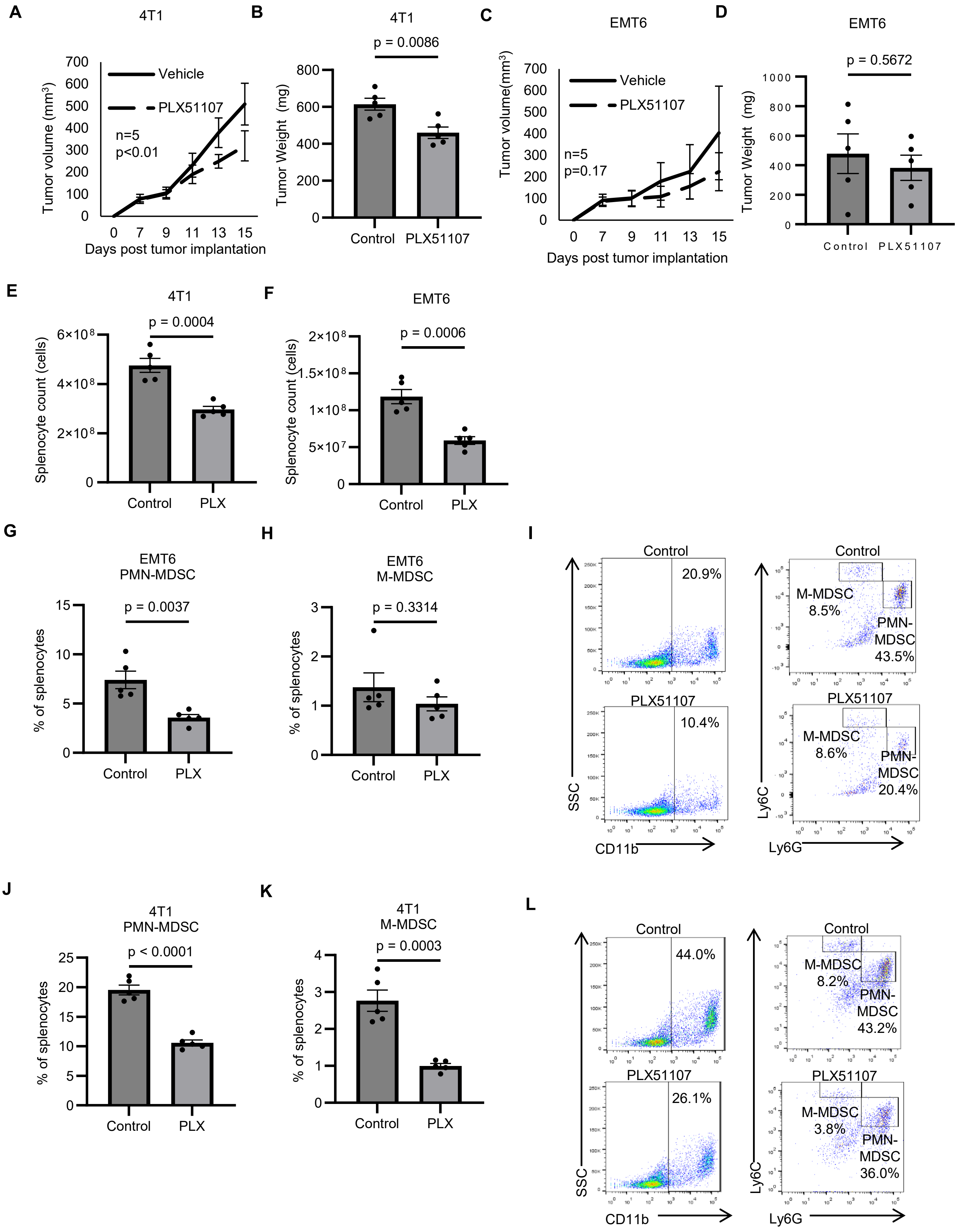
A
4T1
Tumor volume (mm³)
Vehicle
PLX51107
n=5
p<0.01
Days post tumor implantation
B
4T1
p = 0.0086
Tumor Weight (mg)
Control
PLX51107
C
EMT6
Tumor volume(mm³)
Vehicle
PLX51107
n=5
p=0.17
Days post tumor implantation
D
EMT6
p = 0.5672
Tumor Weight (mg)
Control
PLX51107
E
4T1
p = 0.0004
Splenocyte count (cells)
Control
PLX
F
EMT6
p = 0.0006
Splenocyte count (cells)
Control
PLX
G
EMT6
PMN-MDSC
p = 0.0037
% of splenocytes
Control
PLX
H
EMT6
M-MDSC
p = 0.3314
% of splenocytes
Control
PLX
I
Control
20.9%
PLX51107
10.4%
SSC
CD11b
Control
M-MDSC
8.5%
PMN-
MDSC
43.5%
PLX51107
M-MDSC
8.6%
PMN-
MDSC
20.4%
Ly6C
Ly6G
J
4T1
PMN-MDSC
p < 0.0001
% of splenocytes
Control
PLX
K
4T1
M-MDSC
p = 0.0003
% of splenocytes
Control
PLX
L
Control
44.0%
PLX51107
26.1%
SSC
CD11b
Control
M-MDSC
8.2%
PMN-
MDSC
43.2%
PLX51107
M-MDSC
3.8%
PMN-
MDSC
36.0%
Ly6C
Ly6G

Figure S2. PLX51107 alters tumor composition and reduces splenic MDSC subsets.

(A-L). Mice with 4T1 or EMT6 tumors were treated as described in **Figure 2A or 2D**.

(A) Values are the mean ± SEM of 4T1 tumor volumes at each time point, $p=0.006$ at day 15.

(B) Weight of 4T1 tumors from mice treated with the control vehicle or PLX51107, $p=0.008$, unpaired 2-tailed Student's t-test.

(C) Values are the mean ± SEM of 4T1 tumor volumes at each time point, $p<0.01$ at day 15.

(D) Weight of EMT6 tumors from mice treated with the control vehicle or PLX51107, $p=0.56$, unpaired 2-tailed Student's t-test.

(E-F) Spleens were processed, and splenocytes were counted. Graph represents means ± SEM of total splenocytes isolated from **(E)** 4T1 tumor-bearing mice or (F) EMT6 tumor-bearing mice before or after treatment, unpaired 2-tailed Student's t-test.

(G-L) Splenocytes were stained with antibodies against CD11b, Ly6C, and Ly6G. Values displayed are means ± SEM of the frequency of PMN **(G)** and M-MDSC **(H)** subsets within the spleen of 5 EMT6 tumor-bearing mice per group. Representative flow cytometry plots from 2 mice are provided **(I**). Values displayed are means ± SEM of the frequency of PMN **(J)** and M-MDSC **(K)** subsets within the spleen of 5 4T1 tumor-bearing mice per group, unpaired 2-tailed Student's t-test. Representative flow cytometry plots from 2 mice are provided.

Figure S3

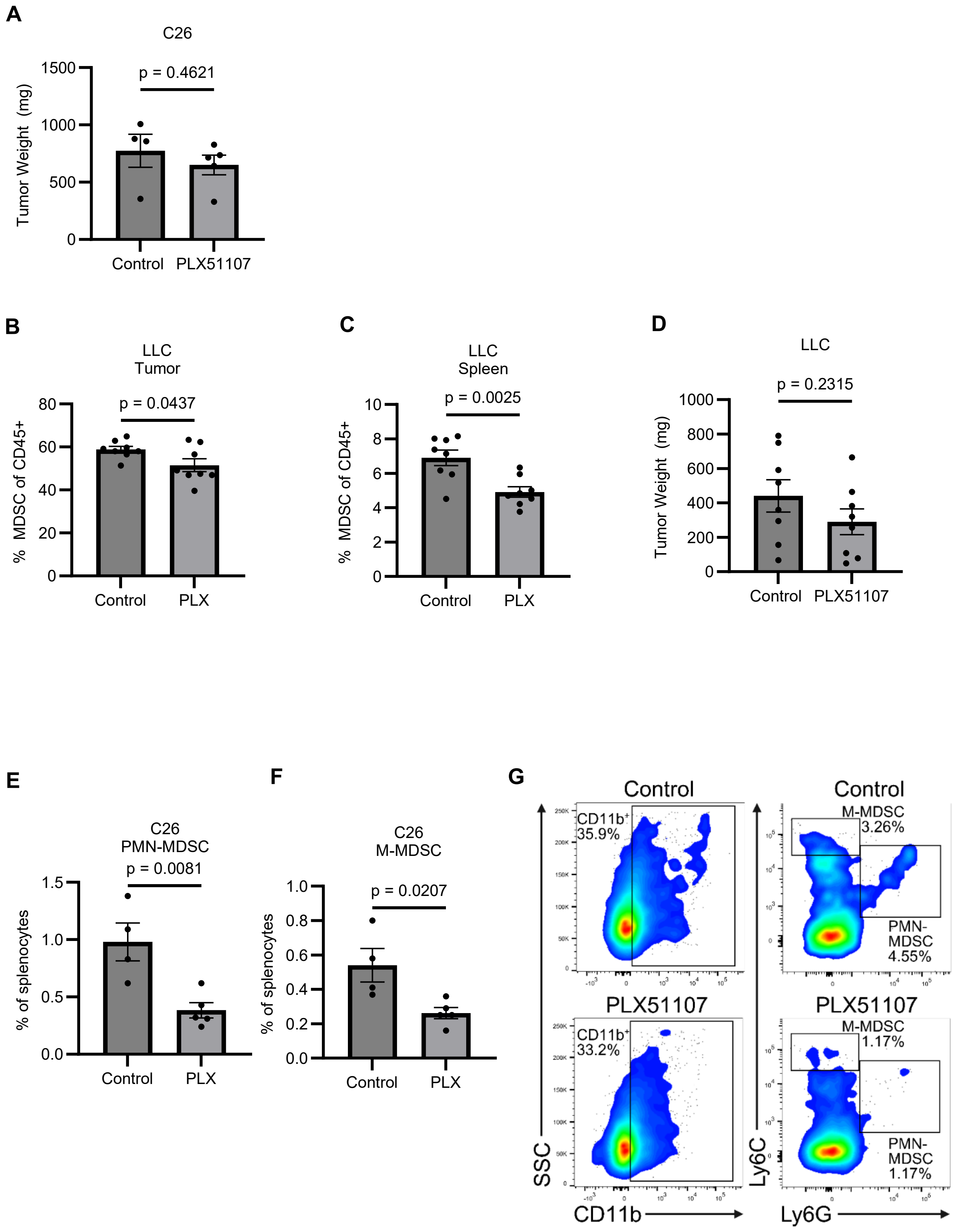
A
C26
p = 0.4621
Tumor Weight (mg)
1500
1000
500
0
Control
PLX51107
B
LLC
Tumor
p = 0.0437
% MDSC of CD45+
80
60
40
20
0
Control
PLX
C
LLC
Spleen
p = 0.0025
% MDSC of CD45+
10
8
6
4
2
0
Control
PLX
D
LLC
p = 0.2315
Tumor Weight (mg)
1000
800
600
400
200
0
Control
PLX51107
E
C26
PMN-MDSC
p = 0.0081
% of splenocytes
1.5
1.0
0.5
0.0
Control
PLX
F
C26
M-MDSC
p = 0.0207
% of splenocytes
1.0
0.8
0.6
0.4
0.2
0.0
Control
PLX
G
Control
CD11b+
35.9%
Control
M-MDSC
3.26%
PMN-
MDSC
4.55%
PLX51107
CD11b+
33.2%
PLX51107
M-MDSC
1.17%
PMN-
MDSC
1.17%
SSC
CD11b
Ly6C
Ly6G

Figure S3. PLX51107 reduces MDSC in C26 and LLC tumor model.

(A) Balb/c mice were inoculated with C26 cells and treated as described in Figure 2I. Values displayed are mean tumor weights ± SEM from 4-5 mice per treatment group, p=0.46, unpaired 2-tailed Student's t-test.

(B-C) C57/black 6 mice were inoculated with LLC cells and treated as described in Figure 2L. Splenocytes and tumors were isolated in single-cell suspension and stained with antibodies against CD45, CD11b, and GR1. Values displayed and means ± SEM of the frequency of MDSC within the tumor **(B)** and spleen **(C)** of 8 tumor-bearing mice per group, unpaired 2-tailed Student's t-test.

(**D**) C57BL/6 mice were inoculated with LLC cells and treated as described in Figure 2L. Values displayed are mean tumor weights ± SEM from 8 mice per treatment group, p=0.23, unpaired 2-tailed Student's t-test..

(E-G) Balb/c mice were inoculated with C26 cells and treated as described in Figure 2I. Splenocytes were then stained with antibodies against CD11b, Ly6G, and Ly6C. Values displayed are means ± SEM of the frequency of PMN **(E)** and M-MDSC **(F)** subsets within the spleen of 5 C26 tumor-bearing mice per group. Representative flow cytometry plots from 2 mice are provided **(G),** unpaired 2-tailed Student's t-test.

Figure S4

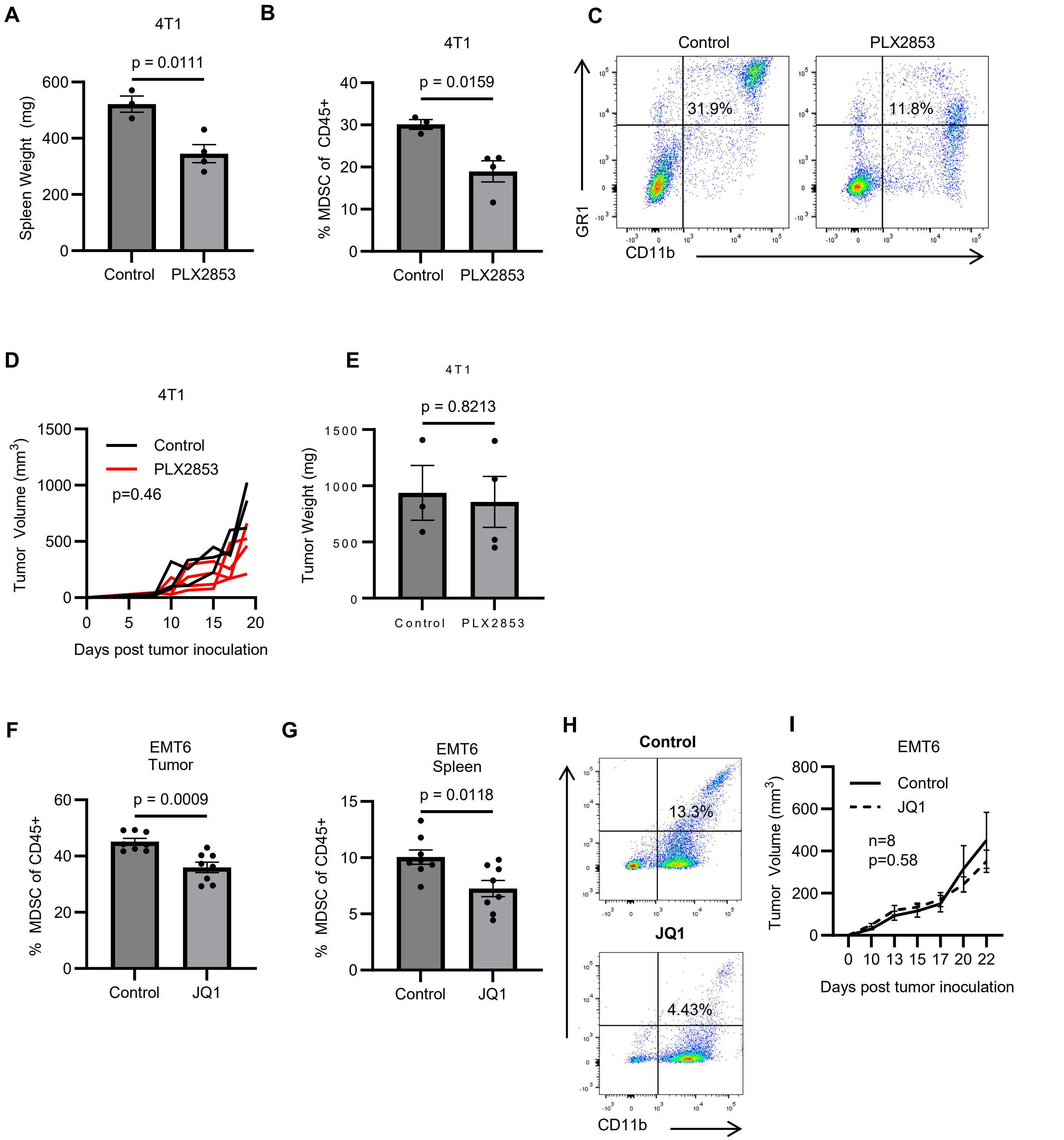
A
4T1
p = 0.0111
Spleen Weight (mg)
Control
PLX2853
B
4T1
p = 0.0159
% MDSC of CD45+
C
Control
PLX2853
31.9%
11.8%
GR1
CD11b
D
4T1
Control
PLX2853
p=0.46
Tumor Volume (mm3)
Days post tumor inoculation
E
4T1
p = 0.8213
Tumor Weight (mg)
F
EMT6
Tumor
p = 0.0009
% MDSC of CD45+
JQ1
G
EMT6
Spleen
p = 0.0118
H
Control
13.3%
JQ1
4.43%
I
EMT6
n=8
p=0.58
Tumor Volume (mm3)
Days post tumor inoculation

Figure S4. The BRD4 inhibitor PLX2853 and JQ1 reduces MDSC in breast cancer models.

(A-E) Balb/c mice were inoculated with $1x10^5$ 4T1 cells, and when tumors were palpable (50 mm3), mice were treated with the control vehicle or 5 mg/kg of PLX2853 daily for 11 days.

(A) Values shown are the mean ± SEM of splenic weights from 3-4 mice per treatment group after 11 days of treatment with the control vehicle or 5 mg/kg PLX2853, $p<0.05$, unpaired 2-tailed Student's t-test.

(B) Values displayed are the mean ± SEM of total GR1+ and CD11b+ MDSC frequency within the spleen of 3-4 mice per treatment group, $p<0.05$, unpaired 2-tailed Student's t-test.

(C) Representative flow cytometry of total MDSC within the spleen from two mice.

(D) Tumor volumes were measured three times weekly with digital calipers. Values are the mean ± SEM of tumor volumes from 3-4 mice per treatment group at each time point, $p=0.46$ at day 19.

(E) Values shown are the mean ± SEM of tumor weights from 3-4 mice per treatment group after 11 days of treatment with the control vehicle or 5 mg/kg PLX2853, $p=0.55$, unpaired 2-tailed Student's t-test.

(F-H) Balb/c mice were injected with $1x10^6$ EMT6 cells in the mammary fat pad. Once tumors were palpable (50 mm^3), mice were treated with DMSO or 50 mg/kg by intraperitoneal injection daily for 12 days. Tumors and spleens were processed and stained with antibodies against GR1 and CD11b to detect total MDSC. Values displayed are the means ± SEM of total $GR1^+$/CD11b+ MDSC within the tumor **(F)** and spleens **(G)** from 8 mice per treatment group, $p<0.001$ in the tumor and $p<0.05$ in the spleen, unpaired 2-tailed Student's t-test.

(H) Representative flow cytometry of total MDSC within the spleen from two mice for **G**.

(I) Tumor volumes were measured three times weekly with digital calipers. Values are the mean ± SEM of tumor volumes from 8 mice per treatment group at each time point, $p=0.58$ at day 22.

Figure S5

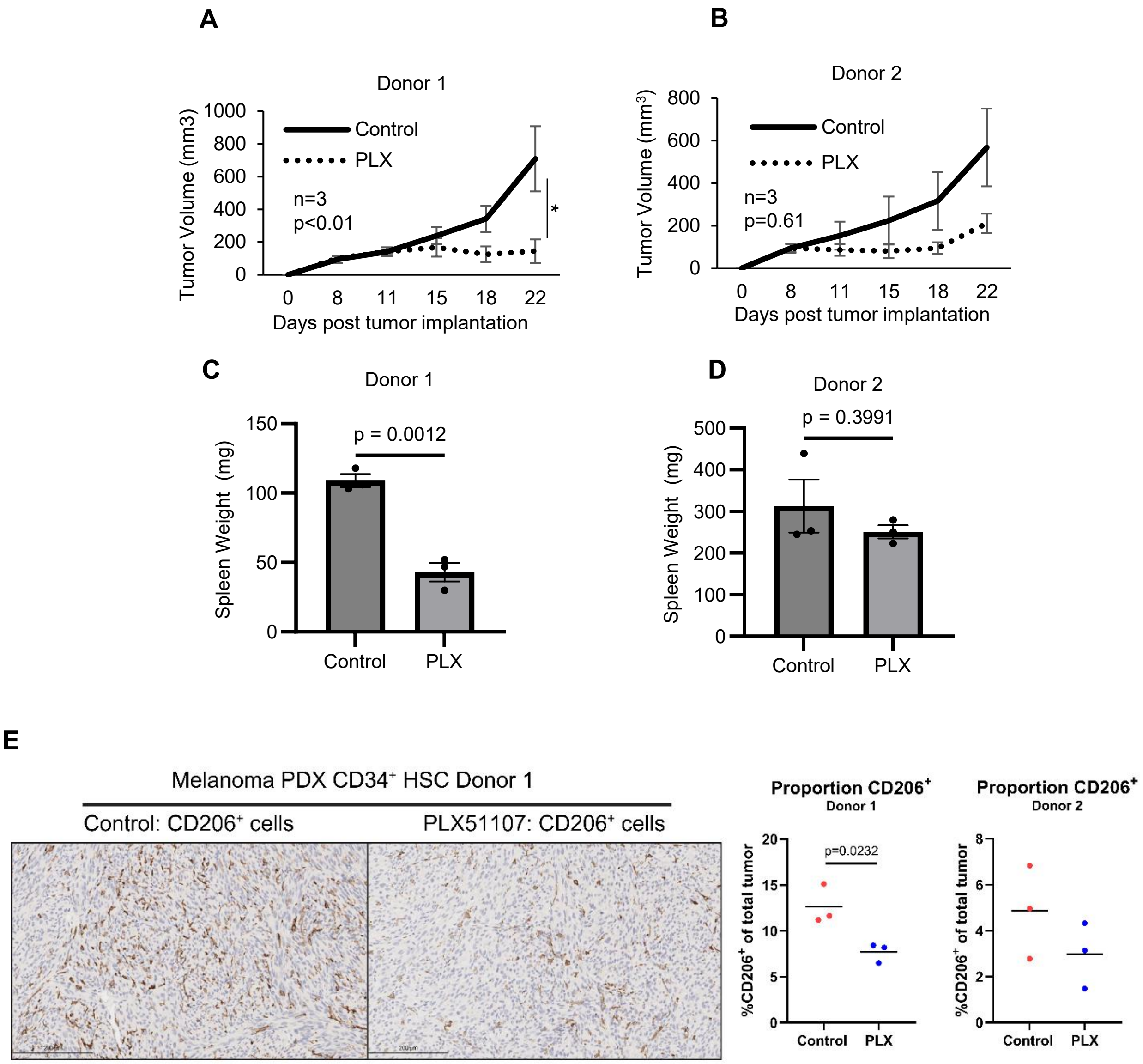

Figure S5. PLX51107 also reduces MDSC in a melanoma PDX model.

CD34+ HSC-engrafted NSG-SGM3 mice were co-engrafted with the melanoma PDX tumor model TM01149. Once tumors were palpable (100m3), mice were treated with control (vehicle) or 20 mg/kg PLX51107 daily via oral gavage. After 15 days of treatment, mice were euthanized. Line graphs represent mean tumor volumes ± SEM. from 3 mice per treatment group for each donor.

(A-B) Tumor volumes were measured three times weekly with digital calipers. Values are the mean ± SEM of tumor volumes from 3 mice per treatment group for each donor at each time point.

(C-D). Spleen weights from mice for each donor, unpaired 2-tailed Student's t-test.

(E). Quantification of melanoma patient-derived xenograft tumor-infiltrating lymphocytes by immunohistochemistry. Representative images of H&E/DAB staining for CD206 in Donor 1. (B) Proportions of CD206⁺ cells of total tumor cells by QuPath quantification, unpaired two-tailed Student's t-test, n=3 per group.

Figure S6

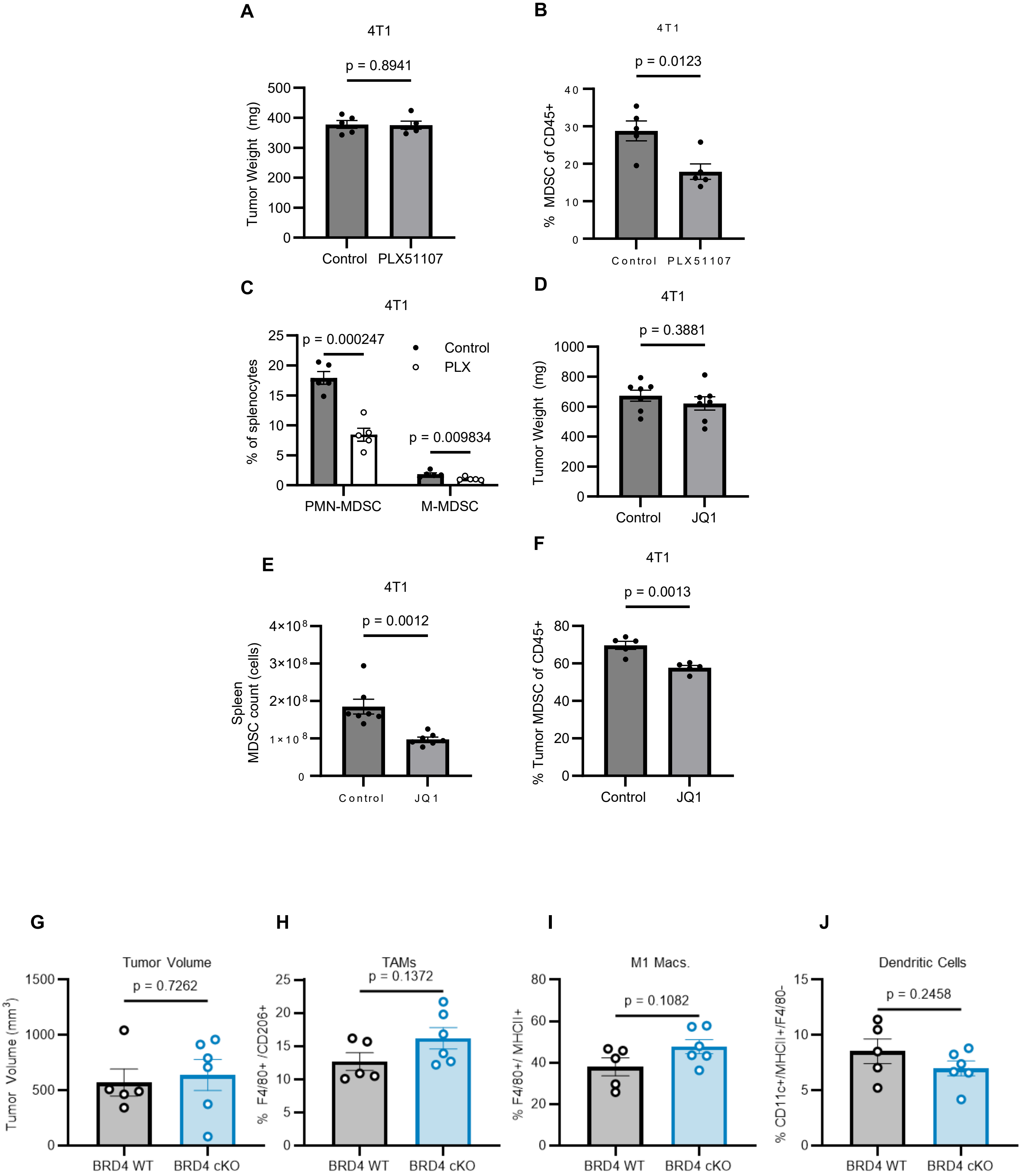

A
4T1
p = 0.8941
Tumor Weight (mg)
500
400
300
200
100
0
Control
PLX51107
B
4T1
p = 0.0123
% MDSC of CD45+
40
30
20
10
0
Control
PLX51107
C
4T1
p = 0.000247
Control
PLX
% of splenocytes
25
20
15
10
5
0
p = 0.009834
PMN-MDSC
M-MDSC
D
4T1
p = 0.3881
Tumor Weight (mg)
1000
800
600
400
200
0
Control
JQ1
E
4T1
p = 0.0012
Spleen
MDSC count (cells)
4×10⁸
3×10⁸
2×10⁸
1×10⁸
0
Control
JQ1
F
4T1
p = 0.0013
% Tumor MDSC of CD45+
80
60
40
20
0
Control
JQ1
G
Tumor Volume
p = 0.7262
Tumor Volume (mm³)
1500
1000
500
0
BRD4 WT
BRD4 cKO
H
TAMs
p = 0.1372
% F4/80+ /CD206+
25
20
15
10
5
0
BRD4 WT
BRD4 cKO
I
M1 Macs.
p = 0.1082
% F4/80+/ MHCII+
80
60
40
20
0
BRD4 WT
BRD4 cKO
J
Dendritic Cells
p = 0.2458
% CD11c+/MHCII+/F4/80-
15
10
5
0
BRD4 WT
BRD4 cKO

Figure S6. Short-term treatment of advanced 4T1 tumors with PLX51107 and JQ1 does not affect tumor volume but still results in reduced MDSC and intratumoral myeloid populations in BRD4 cKO mice vs. WT mice.

(A-C) Balb/c mice were inoculated with $1x10^5$ 4T1 cells in the mammary fat. After two weeks when the mean tumor volume was 200 mm^3 mice were divided into groups and treated for 5 days with the control vehicle or 20 mg/kg PLX51107.
(A) Mean tumor weight ± SEM from 5 mice per treatment group, p=0.89, unpaired 2-tailed Student's t-test.
(B) Splenocytes were stained with antibodies against GR1 and CD11b to detect total MDSC. Values displayed are means ± SEM of the frequency of total MDSC within the spleens of 5 mice per treatment group, p<0.05, unpaired 2-tailed Student's t-test.
(C) Splenocytes were also stained with antibodies against CD11b, Ly6G, and Ly6C to detect PMN and M-MDSC subsets. Values displayed are means ± SEM of each MDSC subset from 5 mice per treatment group, p<0.01 for the PMN subset and p<0.05 for the M-MDSC subset, multiple unpaired 2-tailed Student's t-test with Tukey's correction.

(D-E) Balb/c mice were injected with $1x10^5$ 4T1 cells in the mammary fat pad. After 2 weeks, when the mean tumor volume was 200 mm3, mice were divided into control and JQ1 treatment groups. Mice were treated with DMSO or 50 mg/kg JQ1 daily by intraperitoneal injection daily for 7 days.
(D) Values displayed are the mean weights ± SEM of 4T1 tumors following 7 days of treatment with DMSO or JQ1, p=0.38, unpaired 2-tailed Student's t-test.
(E) Absolute MDSC count within the spleen of 4T1 tumor-bearing mice treated with the DMSO or JQ1. Absolute MDSC count was calculated by multiplying total cells within the spleen (counted using Z Series Coulter Counter) by frequency of MDSC measured by flow cytometry. Bar graphs represent means ± SEM from 7 mice per treatment group, p<0.01, unpaired 2-tailed Student's t-test.

(F) Mice were treated as in **Figure S6D**. After 7 days of treatment, tumors were processed into single-cell suspensions and stained with antibodies against CD45, GR1, and CD11b. Values displayed are the means ± SEM of the frequency of GR1+ and CD11b+ MDSC within the CD45+ fraction of cells within the tumors from 5 mice per treatment group, p<0.01, unpaired 2-tailed Student's t-test.

(G) LLC tumor volume in BRD4 WT and BRD4 cKO mice.

(H-J) Frequency of tumor associated macrophages (F4/80+ CD206+), M1-like macrophages (F4/80, MHCII), dendritic cells (CD11c+, MHCII+, F4/80-) within LLC tumors of BRD4 WT or BRD4 cKO mice were measured by flow cytometry. Bar graphs represent mean ±SEM from 5-6 mice per group, unpaired 2-tailed Student's t-test.

Figure S7

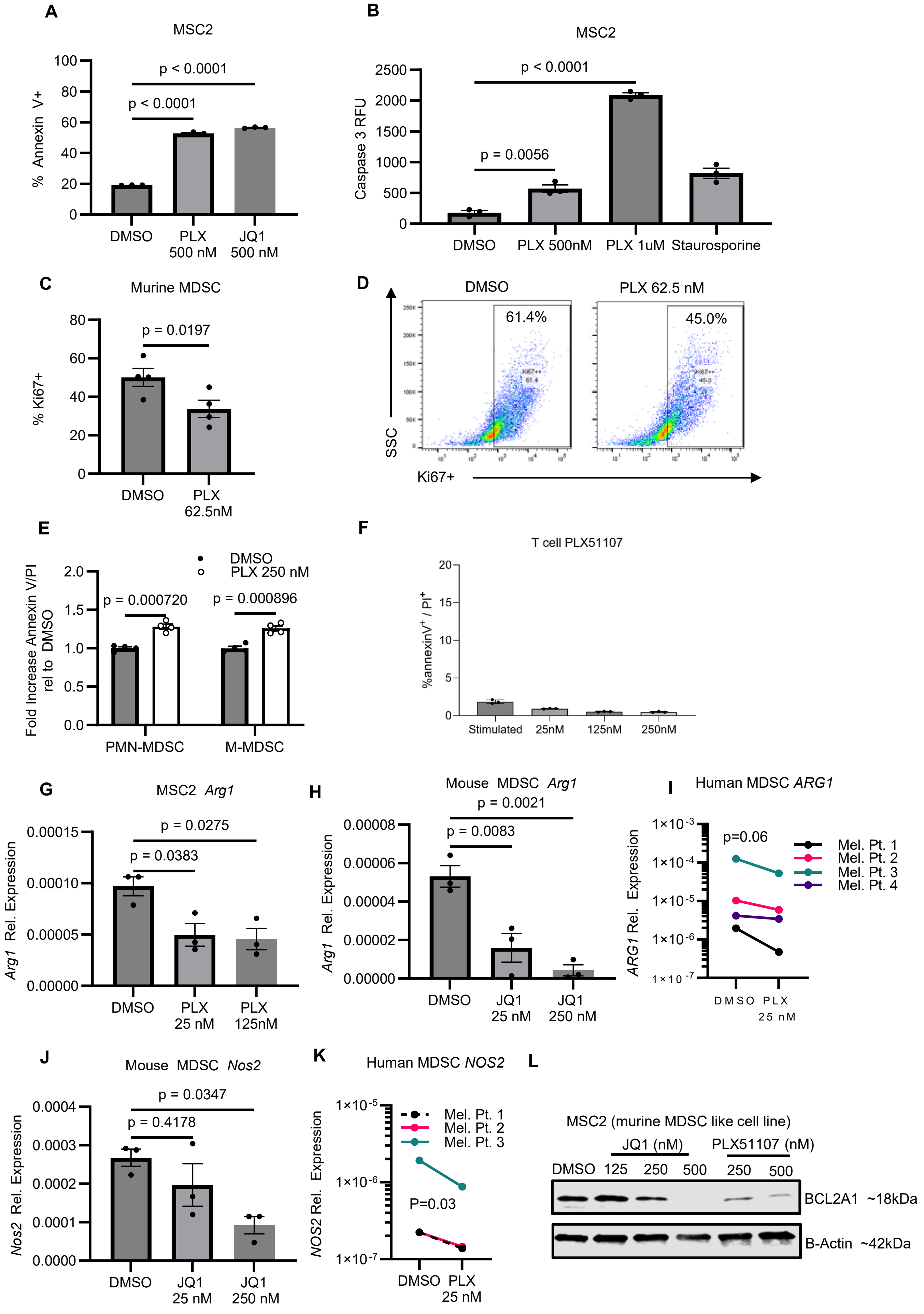
A
MSC2
% Annexin V+
p < 0.0001
p < 0.0001
DMSO
PLX 500 nM
JQ1 500 nM
B
MSC2
Caspase 3 RFU
p < 0.0001
p = 0.0056
DMSO
PLX 500nM
PLX 1uM
Staurosporine
C
Murine MDSC
% Ki67+
p = 0.0197
DMSO
PLX 62.5nM
D
DMSO
PLX 62.5 nM
61.4%
45.0%
SSC
Ki67+
E
DMSO
PLX 250 nM
Fold Increase Annexin V/PI rel to DMSO
p = 0.000720
p = 0.000896
PMN-MDSC
M-MDSC
F
T cell PLX51107
%annexinV+ / PI+
Stimulated
25nM
125nM
250nM
G
MSC2 Arg1
Arg1 Rel. Expression
p = 0.0275
p = 0.0383
DMSO
PLX 25 nM
PLX 125nM
H
Mouse MDSC Arg1
Arg1 Rel. Expression
p = 0.0021
p = 0.0083
DMSO
JQ1 25 nM
JQ1 250 nM
I
Human MDSC ARG1
ARG1 Rel. Expression
p=0.06
Mel. Pt. 1
Mel. Pt. 2
Mel. Pt. 3
Mel. Pt. 4
DMSO
PLX 25 nM
J
Mouse MDSC Nos2
Nos2 Rel. Expression
p = 0.0347
p = 0.4178
DMSO
JQ1 25 nM
JQ1 250 nM
K
Human MDSC NOS2
NOS2 Rel. Expression
Mel. Pt. 1
Mel. Pt. 2
Mel. Pt. 3
P=0.03
DMSO
PLX 25 nM
L
MSC2 (murine MDSC like cell line)
JQ1 (nM)
PLX51107 (nM)
DMSO
125
250
500
250
500
BCL2A1 ~18kDa
B-Actin ~42kDa

Figure S7. Brd4 inhibition reduces MDSC proliferation and induces apoptosis.

(A) MSC2 cells were treated with DMSO as a control, PLX51107 (500 nM), or JQ1 (500 nM) for 24 hours. After 24 hours, cells were collected and stained with annexin and PI to detect apoptosis. Data displayed represents the mean ± SEM of annexin positive cells from three independent experiments, $p<0.0001$ for DMSO vs 500 nM PLX51107, $p<0.0001$ for DMSO vs. 500 nM JQ1, One-way ANOVA model with Tukey's correction.

(B) MSC2 cells were treated with DMSO as a negative control, staurosporine as a positive control or the indicated dose of PLX51107 for 24 hours and then caspase 3 activity was measured with a DEVD-AFC fluorometric assay. Values displayed are means ± SEM of relative fluorescence units from three independent experiments, $p<0.01$ for DMSO vs 500nM, and $p<0.0001$ for DMSO vs. 1000 nM PLX51107, One-way ANOVA model with Tukey's correction.

(C) Splenic murine MDSC isolated from 4T1 tumor-bearing mice were stimulated *in vitro* with 100 ng/mL IL-6 and GM-CSF for 72h in media containing DMSO, or 62.5 nM PLX51107. Ki67+ MDSC was assessed via flow cytometry. Values displayed are means ± SEM of %Ki67 MDSC. from 4 independent experiments, $p<0.05$ for DMSO vs. PLX 62.5 nM, unpaired 2-tailed Student's t-test.

(D) Representative flow cytometry plots for Ki67+ staining in **C**.

(E) PMN and M-MDSC subsets were isolated from the spleens of 4T1 tumor-bearing mice and then treated with DMSO or 250 nM PLX51107 for 12 hours. After 12 hours, cells were collected and stained with annexin and PI to detect apoptotic cells. Values displayed are fold change ± SEM of the frequency of annexin positive cells from four independent experiments, $p<0.001$ for both MDSC subsets, multiple unpaired 2-tailed Student's t-test with Tukey's correction.

(F) $CD3^+$ T cells were isolated from spleens of healthy BALB/c mice and stimulated with anti-CD3/CD28 beads for 48 hours. Cells were cultured with increasing concentrations of PLX51107 or DMSO vehicle control and stained for annexinV and PI. Values represent proportion annexin V^+ PI^+ cells, n=3, one-way ANOVA with Tukey correction.

(G) MSC2 cells were treated for 24h with DMSO control or indicated dose of PLX51107. Relative gene expression of *ARG1* across treatment group normalized to *B-actin*, $p<0.05$ DMSO vs. PLX 25 nM, and DMSO vs. PLX 250 nM, One-way ANOVA model with Tukey's correction.

.

(H) Splenic murine MDSC were isolated from 4T1 tumor-bearing mice treated for 24h with DMSO control or indicated dose of PLX51107 with media supplemented with 10 ng/ml IL-6 and GM-CSF. Relative gene expression of *ARG1* across the treatment group normalized to *B-actin*, $p<0.01$ for DMSO vs. PLX 25 nM, and $p<0.01$ for DMSO vs. PLX 250 nM , One-way ANOVA model with Tukey's correction.

(I) $CD33^+/CD11b^+/HLADR^{lo/-}$ MDSCs were isolated from the peripheral blood of patients with melanoma by FACS. Cells were cultured in HAB media supplemented with 10 ng/ml IL-6 and GM-CSF and treated with DMSO or the indicated concentration of PLX51107. After 24 hours RNA was isolated from cells and relative gene expression of *ARG1* across treatment group normalized to *B-actin*, $p=0.06$ for DMSO vs. PLX 25 nM, paired 2-tailed Student's t-test.

(J) Splenic murine MDSC were isolated from 4T1 tumor-bearing treated as in **H**. Relative gene expression of *NOS2* across treatment group normalized to *B-actin*, $p<0.05$ for DMSO vs. PLX 250 nM, One-way ANOVA model with Tukey's correction.

(K) $CD33^+/CD11b^+/HLADR^{lo/-}$ MDSCs were isolated from the peripheral blood of patients with melanoma by FACS and treated as in **I. R**elative gene expression of *NOS2* across treatment group normalized to *B-actin*, $p<0.05$ for DMSO vs. PLX 25 nM, paired 2-tailed Student's t-test.

(L) MSC2 cells treated for 24h with DMSO control or indicated dose of PLX51107 or JQ1. Protein lysates of treated cells were probed with antibodies for BCL2A1 and β -actin.

Table 1. Differential expression EMT6 model

Gene	Fold change	p value
Ccr2	0.18	3.21E-12
Il24	0.23	1.99E-08
S100a8	0.24	7.63E-09
Ccr5	0.32	6.41E-11
Ccl12	0.35	1.05E-05
Cxcl5	0.35	1.78E-03
Epcam	0.36	2.19E-02
Il23a	0.38	5.50E-04
Il1b	0.38	3.79E-08
Cxcl3	0.38	4.73E-03
Nlrp3	0.40	1.16E-06
Rorc	0.40	2.71E-03
Ccl24	0.40	7.20E-03
Tlr6	0.41	1.64E-08
Ccr1	0.41	1.21E-09
Il34	0.43	1.30E-09
Cd180	0.45	5.45E-06
Ifna1	0.46	2.88E-02
Il6	0.46	3.88E-04
Mmp9	0.47	9.30E-03
Tlr1	0.48	3.02E-07
Csf3r	0.49	2.91E-05
Lif	0.50	1.93E-06
Gata3	0.50	4.72E-02
Gpr183	0.50	1.56E-04
Tnfsf10	0.51	3.91E-04
F2rl1	0.51	1.18E-02
Cdh1	0.52	4.28E-02
Csf3	0.52	1.92E-02
Mefv	0.52	1.99E-03
Cd80	0.53	3.77E-05
Tlr4	0.53	4.05E-06
Sbno2	0.53	2.14E-06
Cma1	0.53	2.70E-03
Il18rap	0.53	1.68E-04
Csf2	0.54	1.15E-03
Il7	0.54	9.44E-04
Plau	0.54	3.64E-07
Tlr3	0.55	9.17E-06

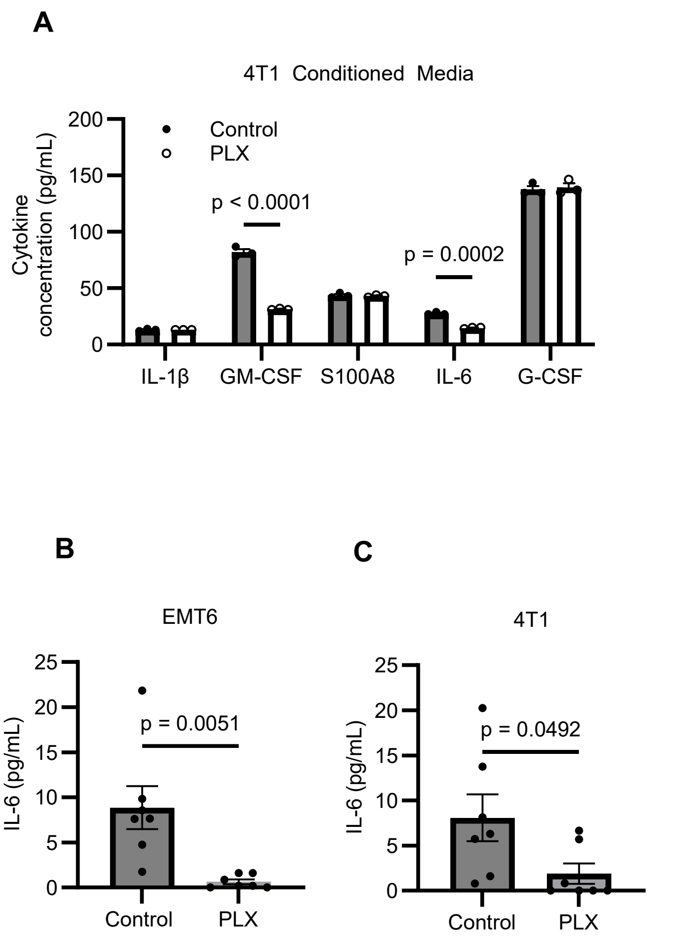

Figure S8. PLX51107 decreases the expression of cytokines that promote MDSC expansion.

(Table 1) Top 40 most down-regulated genes in EMT6 tumors treated with 20 mg/kg PLX51107 for one week compared to control treated mice as measured using the Nanostring PanCancer immune profiling panel.

(A) 4T1 cells were treated with DMSO or 250 nM PLX51107 for 24 hours. Supernatants were then collected, and cytokine expression was analyzed via Luminex multiplex cytokine assay. Bar graphs represent means ± SEM of cytokine concentration from three independent experiments. GM-CSF decreased 2.6-fold, $p<0.0001$ and IL-6 decreased 1.9-fold, $p<0.001$, two-way ANOVA model with Tukey's correction.

(B-C) ELISA measurement of IL-6 protein in the serum of mice with EMT6 **(B)** or 4T1 **(C)** tumors treated with control or 20 mg/kg PLX51107. Values shown are means ± SEM from 7 mice per treatment group, $p<0.01$ for EMT6 and $p<0.05$ for 4T1, unpaired 2-tailed Student's t-test.

Figure S9

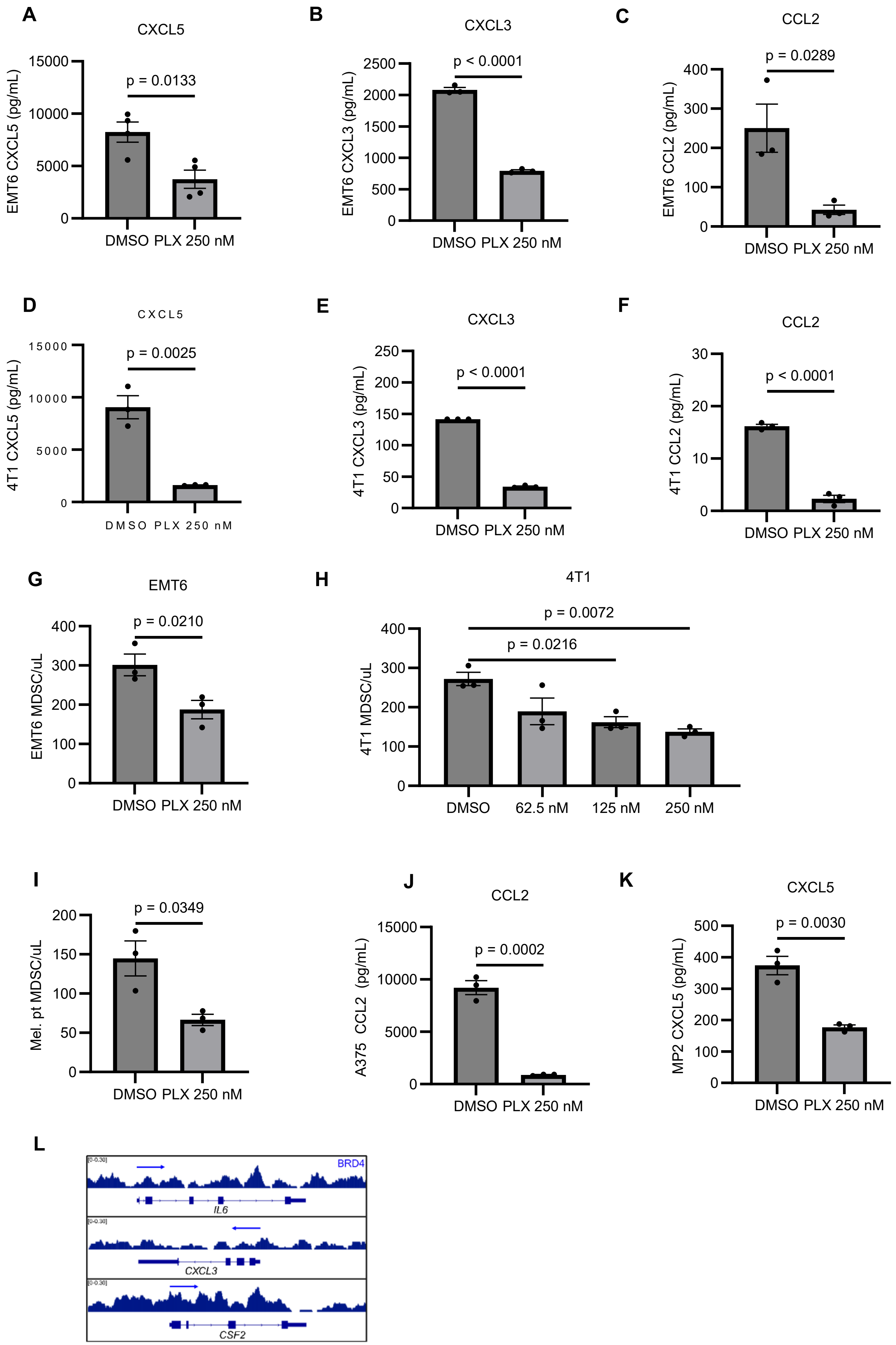
A
CXCL5
EMT6 CXCL5 (pg/mL)
p = 0.0133
DMSO PLX 250 nM
B
CXCL3
EMT6 CXCL3 (pg/mL)
p < 0.0001
DMSO PLX 250 nM
C
CCL2
EMT6 CCL2 (pg/mL)
p = 0.0289
DMSO PLX 250 nM
D
CXCL5
4T1 CXCL5 (pg/mL)
p = 0.0025
DMSO PLX 250 nM
E
CXCL3
4T1 CXCL3 (pg/mL)
p < 0.0001
DMSO PLX 250 nM
F
CCL2
4T1 CCL2 (pg/mL)
p < 0.0001
DMSO PLX 250 nM
G
EMT6
EMT6 MDSC/uL
p = 0.0210
DMSO PLX 250 nM
H
4T1
4T1 MDSC/uL
p = 0.0072
p = 0.0216
DMSO
62.5 nM
125 nM
250 nM
I
Mel. pt MDSC/uL
p = 0.0349
DMSO PLX 250 nM
J
CCL2
A375 CCL2 (pg/mL)
p = 0.0002
DMSO PLX 250 nM
K
CXCL5
MP2 CXCL5 (pg/mL)
p = 0.0030
DMSO PLX 250 nM
L
BRD4
IL6
CXCL3
CSF2

Figure S9. PLX51107 inhibits MDSC migration and chemokine expression by tumor cells.

Conditioned media was generated by plating 2x10^5 EMT6, 4T1, of A375 cells in 24 well plates and treating them with DMSO or the indicated dose of PLX51107 for 24 hours. After 24 hours, the conditioned media was harvested and used in migration assays or for ELISAs to measure chemokine concentration.

(A-F) ELISA measurement of CXCL5, CXCL3, and CCL2 in the conditioned media generated as above from EMT6 **(A-C)** and 4T1 **(D-F)** cells treated with DMSO or 250 nM PLX51107. Values shown are means ± SEM from 3 independent experiments, $p<0.01$ for all comparisons, unpaired 2-tailed Student's t-test.

(G-H) Conditioned media from DMSO or PLX51107 treated EMT6 **(G)** or 4T1 **(H)** cells was used as the stimulus for the migration of MDSC isolated from the spleens of EMT6 or 4T1 tumor-bearing mice using Transwell migration assays. After 12 hours, migrated cells were harvested and quantified by flow cytometry using CountBright cell counting beads. Values shown are means ± SEM of migrated cells from 3 independent experiments. EMT6, $p<0.05$ unpaired 2-tailed Student's t-test. 4T1, $p<0.05$ or $p<0.01$ where indicated, one-way ANOVA model with Tukey's correction.

(I) Migration assays as described above using conditioned media from A375 melanoma cells and MDSC isolated from the peripheral blood of melanoma patients by FACs. Values shown are means ± SEM of migrated cells from 3 independent experiments, $p<0.05$, unpaired 2-tailed Student's t-test.

(J) ELISA measurement of CCL2 protein in conditioned media from A375 cells treated with DMSO or 250 nM of PLX51107. Values shown are means ± SEM from 3 independent experiments, $p<0.001$, unpaired 2-tailed Student's t-test.

(K) ELISA measurement of CXCL5 protein in conditioned media from Mia-PaCa2 (MP2) cells treated with DMSO or 250 nM of PLX51107. Values shown are means ± SEM from 3 independent experiments, $p<0.01$, unpaired 2-tailed Student's t-test.

(L) BRD4 ChIP-seq of MDSC-recruiting soluble factors in the MDA-MB-231 TNBC human cell line determined from a public dataset (42).

Figure S10

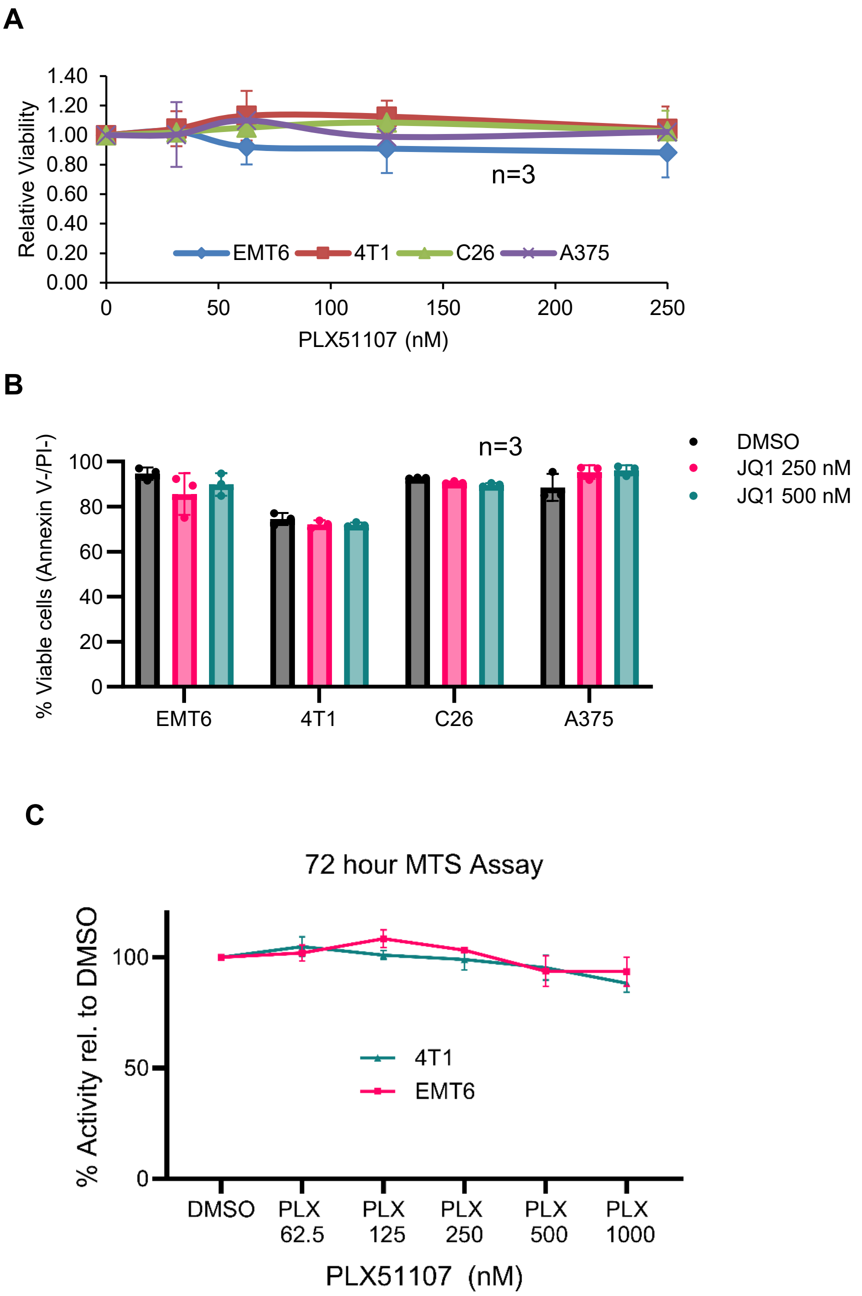

Figure S10. PLX51107 does not affect cancer cell line viability.

(A) Indicated cell lines were plated in triplicate per treatment in 96 well plated and treated with DMSO as a control, 31.25 nM, 62.5 nM, 125 nM, or 250 nM of PLX51107 for 24 hours. After 24 hours MTS assay was used to assess the metabolic activity of the cells, and relative viability was assessed against the DMSO control group. Values displayed are the means ± SEM from three independent experiments per cell line.

(B) Indicated cell lines were plated in 24 well plates and treated with DMSO as a control 250 nM or 500 nM PLX51107 for 24 hours. After 24 hours cells were collected and stained with annexin and PI to detect apoptosis. Values displayed are the means ± SEM of the frequency of annexin negative and PI negative viable cells from 3 independent experiments per cell line

(C) Effect of PLX51107 on cancer cell line viability. Indicated cell lines were plated in triplicate per treatment in 96 well plated and treated with DMSO as a control and indicated concentration of PLX51107 for 72 hours. After 72 hours MTS assay was used to assess the viability activity of the cells, and relative viability was assessed against the DMSO control group. Values displayed are the means ± SEM from three independent experiments per cell line.

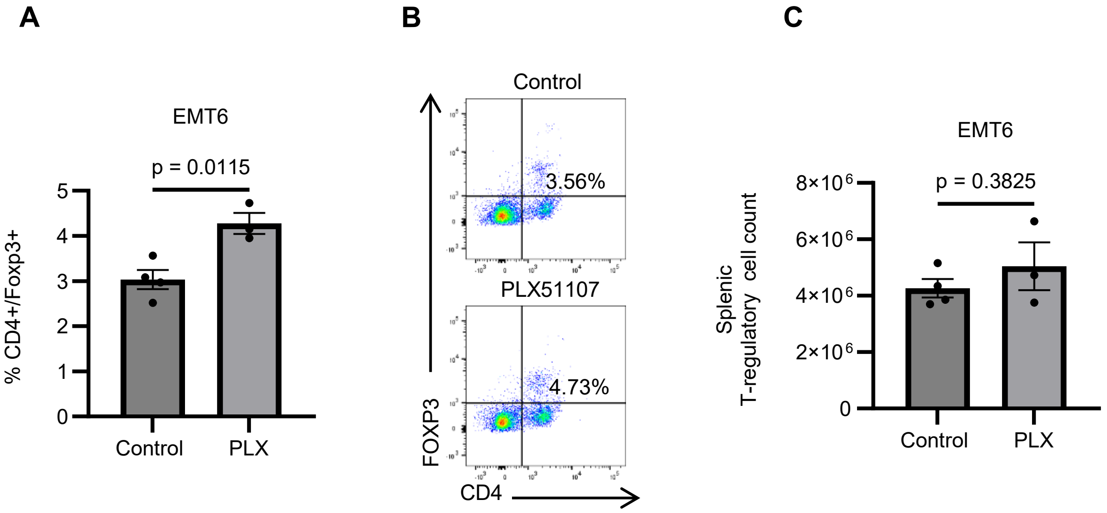

Figure S11. PLX51107 does not significantly alter the number of Tregs within the spleen.

Mice with EMT6 tumors were treated as described in Figure 1. Splenocytes were then stained with antibodies against CD4 and intracellular FOXP3 to detect regulatory T cells.

(A) Values displayed are means ± SEM of the frequency of regulatory T cells within the spleen of three mice per group. $p<0.05$, unpaired 2-tailed Student's t-test.

(B) Representative flow cytometry plot of **A.**

(C) Absolute Treg count within the spleen of EMT6 tumor-bearing mice treated with the DMSO or PLX51107. Absolute Treg count was calculated by multiplying total cells within the spleen (counted using Z Series Coulter Counter) by the frequency of Treg measured by flow cytometry. Bar graphs represent means ± SEM from three mice per treatment group, $p=0.38$, unpaired 2-tailed Student's t-test.

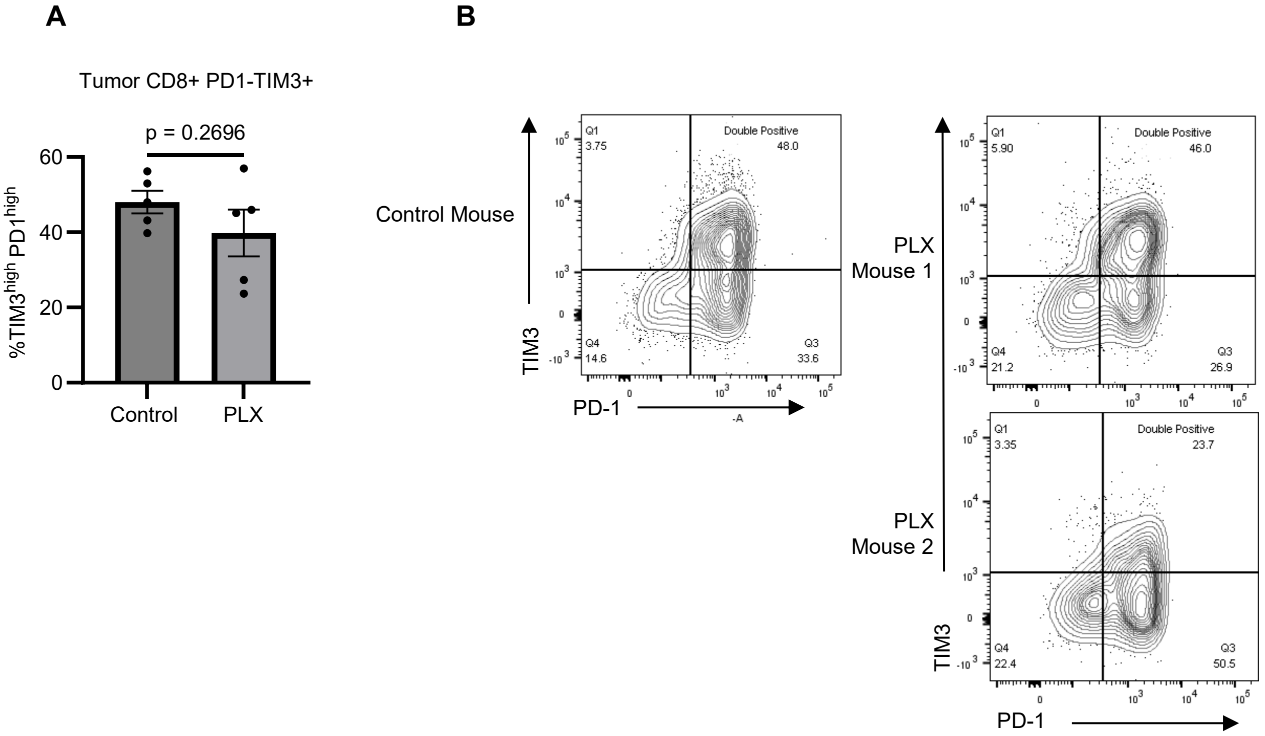

Figure S12. PLX51107 does not significantly alter exhausted CD8 T cells in tumor.

4T1 tumor-bearing mice were treated with control or PLX51107 (20mg/kg) for 12 days. The tumors were processed into a single-cell suspension and stained with the antibodies CD8, TIM3, and PD1.

(A) Values displayed are the means ± SEM of $CD8^+$ T cell expression of TIM3 and PD1 from 5 mice per treatment group, p=0.26, unpaired 2-tailed Student's t-test.

(B) Representative flow cytometry plots of 3 mice.

Figure S13

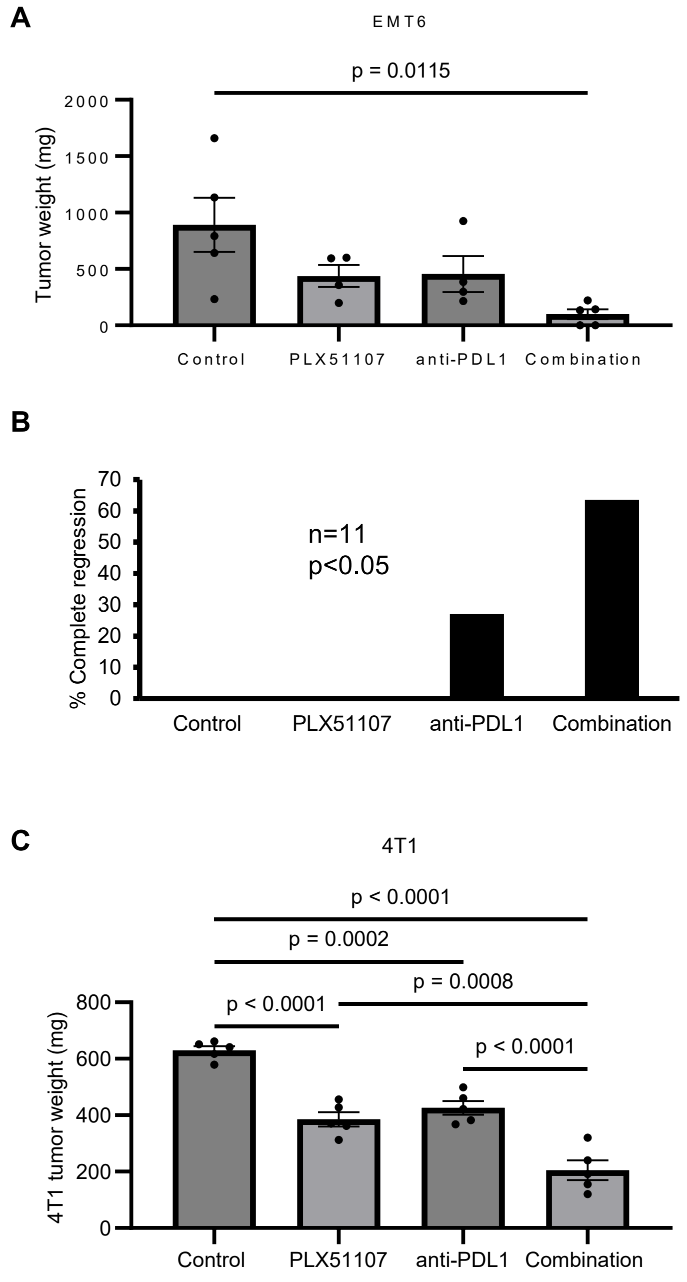

Figure S13. The combination of PLX51107 and anti-PDL1 significantly reduces tumor weight and increases the frequency of complete tumor regression.

(A) Balb/c mice were injected with EMT6 cells and treated as described in Figure 6. Values displayed are the means ± SEM of tumor weights from 4-5 mice per treatment group, $p<0.05$ for control vs combination, one-way ANOVA model with Tukey's correction.

(B) Frequency of complete regression of EMT6 tumors from each treatment group: 0/11 control, 0/11 PLX51107, 3/11 anti-PDL1, and 7/11 combination. $p<0.05$ from anti-PDL1 vs combination group.

(C) Balb/c mice were injected with 4T1 cells and treated as described in Figure 6. Values displayed are the means ± SEM of tumor weights from 5 mice per treatment group, $p<0.001$ for PLX51107 vs combination and $p<0.0001$ anti-PDL1 vs combination, one-way ANOVA model with Tukey's correction.

Figure S14

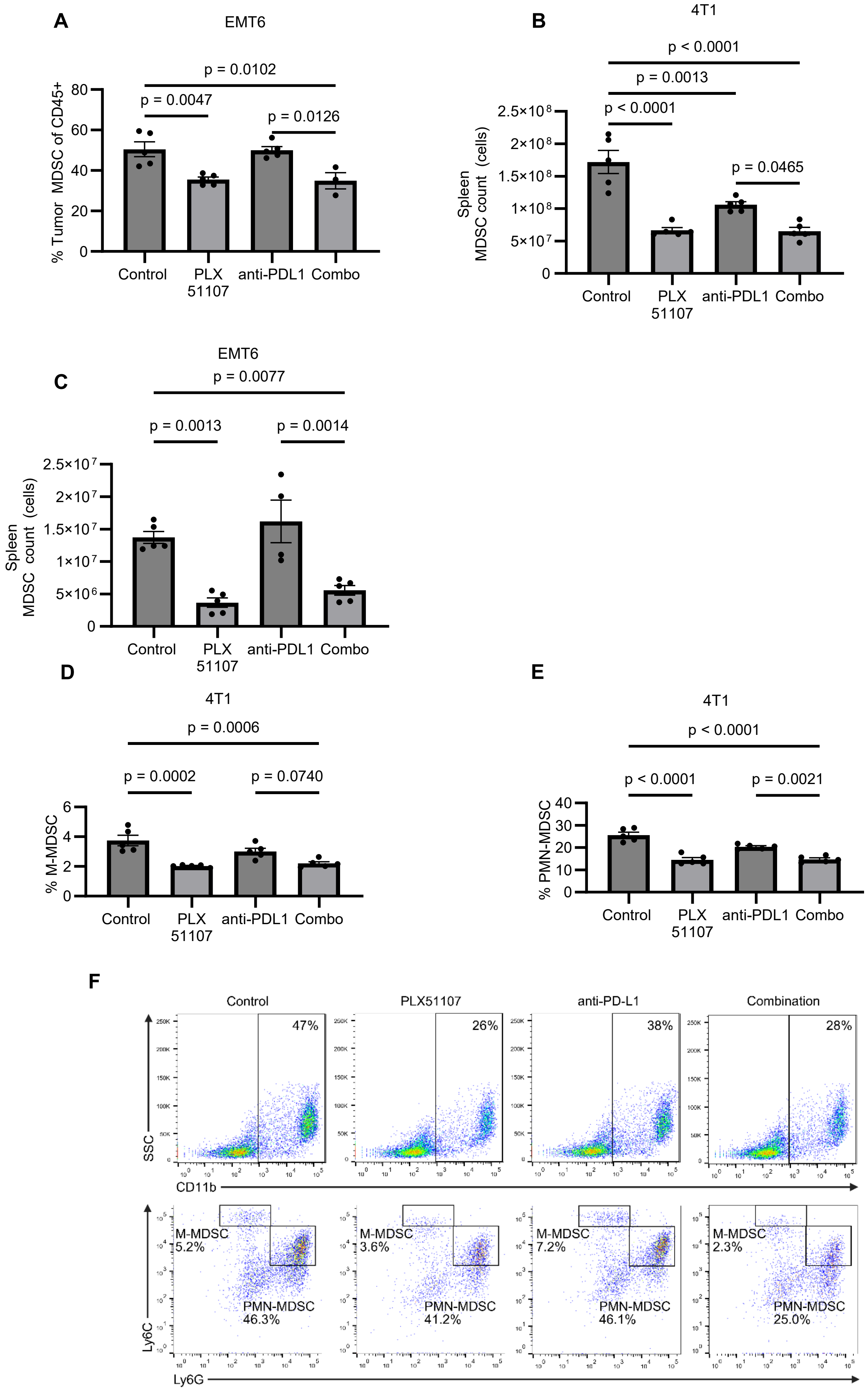

A
EMT6
p = 0.0102
p = 0.0047
p = 0.0126
% Tumor MDSC of CD45+
80
60
40
20
0
Control
PLX 51107
anti-PDL1
Combo
B
4T1
p < 0.0001
p = 0.0013
p < 0.0001
p = 0.0465
Spleen MDSC count (cells)
2.5×10^8
2×10^8
1.5×10^8
1×10^8
5×10^7
0
Control
PLX 51107
anti-PDL1
Combo
C
EMT6
p = 0.0077
p = 0.0013
p = 0.0014
Spleen MDSC count (cells)
2.5×10^7
2×10^7
1.5×10^7
1×10^7
5×10^6
0
Control
PLX 51107
anti-PDL1
Combo
D
4T1
p = 0.0006
p = 0.0002
p = 0.0740
% M-MDSC
6
4
2
0
Control
PLX 51107
anti-PDL1
Combo
E
4T1
p < 0.0001
p < 0.0001
p = 0.0021
% PMN-MDSC
40
30
20
10
0
Control
PLX 51107
anti-PDL1
Combo
F
Control
PLX51107
anti-PD-L1
Combination
47%
26%
38%
28%
SSC
CD11b
M-MDSC 5.2%
PMN-MDSC 46.3%
M-MDSC 3.6%
PMN-MDSC 41.2%
M-MDSC 7.2%
PMN-MDSC 46.1%
M-MDSC 2.3%
PMN-MDSC 25.0%
Ly6C
Ly6G

Figure S14.

(A) EMT6 tumors from **Figure 6C** were harvested and processed into single-cell suspensions and then stained with antibodies against CD45, GR1, and CD11b. Values depicted are means ± SEM of GR1+/CD11b+ MDSC within the CD45+, $p<0.01$ for control vs PLX51107 and $p<0.05$ for anti-PDL1 vs the combination treatment, one-way ANOVA model with Tukey's correction.

(B) 4T1 Absolute MDSC counts were calculated by multiplying total cells within the spleen (counted using Z Series Coulter Counter) by the frequency of MDSCs measured by flow cytometry from **Figure 6E**. Bar graphs represent means ± SEM from 5 mice per treatment group, $p<0.0001$ for control vs. PLX51107 and $p<0.05$ for anti-PDL1 vs. combination, one-way ANOVA model with Tukey's correction.

(C) EMT6 Absolute MDSC counts were calculated by multiplying total cells within the spleen (counted using Z Series Coulter Counter) by the frequency of MDSC measured by flow cytometry from **Figure 6C**. Bar graphs represent means ± SEM from 5 mice per treatment group, $p<0.01$ for control vs. PLX51107 and $p<0.01$ for anti-PDL1 vs. combination, one-way ANOVA model with Tukey's correction.

(D-E). Splenocytes from 4T1 tumor-bearing mice treated as described in **Figure 6E** were stained with antibodies against CD11b, Ly6G, and Ly6C to identify PMN and M-MDSC subsets. Values shown are means ± SEM of the frequency of each MDSC subset within the spleen, one-way ANOVA model with Tukey's correction.

(F) Representative flow cytometry plots showing the frequency of PMN and M-MDSC subsets within the spleen of a 4T1 tumor-bearing mouse from each treatment group

Figure S15

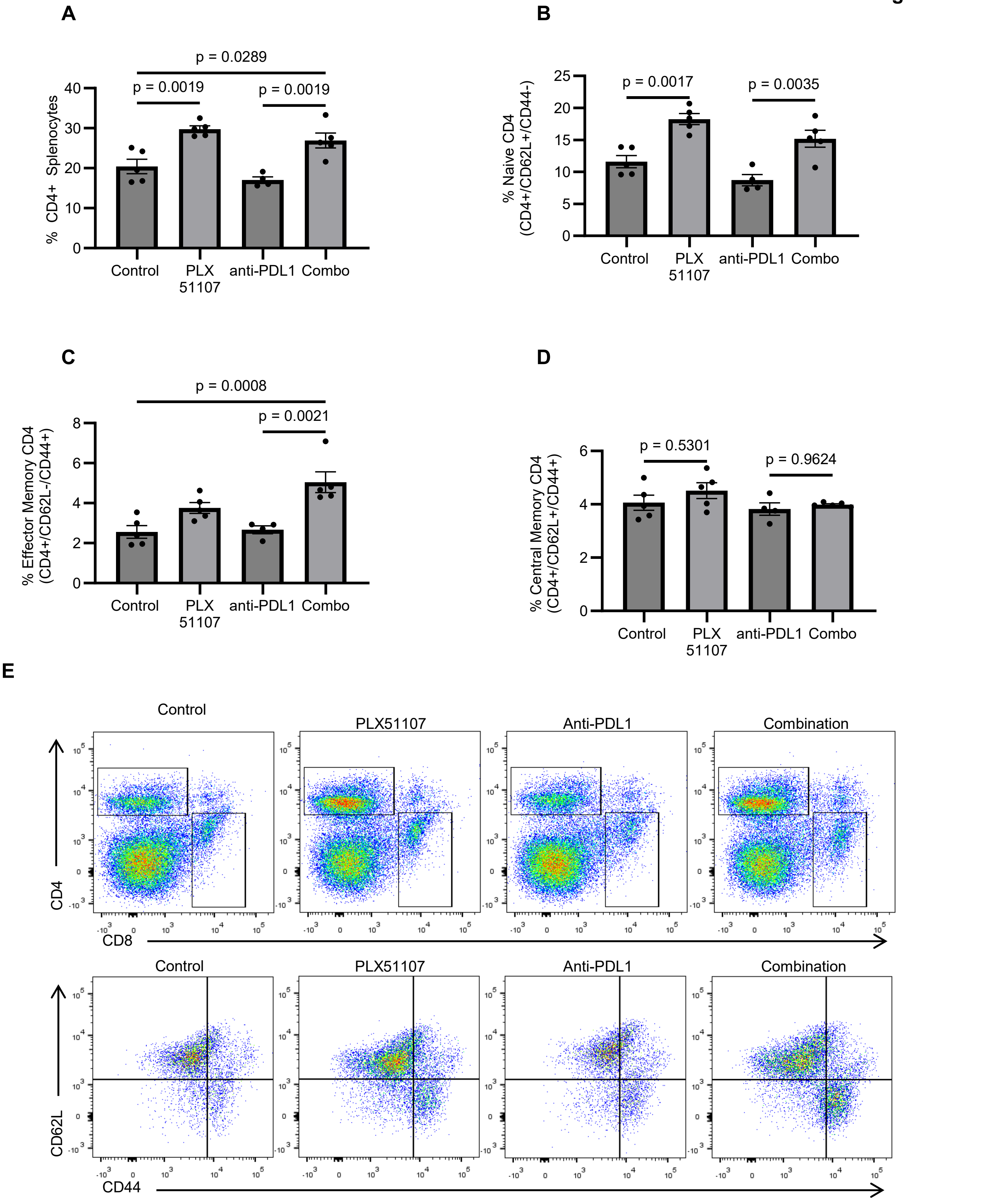

A
p = 0.0289
p = 0.0019
p = 0.0019
% CD4+ Splenocytes
40
30
20
10
0
Control
PLX 51107
anti-PDL1
Combo
B
p = 0.0017
p = 0.0035
% Naive CD4 (CD4+/CD62L+/CD44-)
25
20
15
10
5
0
Control
PLX 51107
anti-PDL1
Combo
C
p = 0.0008
p = 0.0021
% Effector Memory CD4 (CD4+/CD62L-/CD44+)
8
6
4
2
0
Control
PLX 51107
anti-PDL1
Combo
D
p = 0.5301
p = 0.9624
% Central Memory CD4 (CD4+/CD62L+/CD44+)
6
4
2
0
Control
PLX 51107
anti-PDL1
Combo
E
Control
PLX51107
Anti-PDL1
Combination
CD4
CD8
Control
PLX51107
Anti-PDL1
Combination
CD62L
CD44

Figure S15. Frequency of $CD4^+$ T cell phenotypes within the spleen in the 4T1 breast cancer model.

Balb/c mice were inoculated with 4T1 cells and treated as described in Figure 6. Splenocytes were stained with antibodies against CD4, CD8, CD62L, and CD44 and analyzed by flow cytometry.

(A) Values displayed are the means ± SEM of the frequency of total $CD4^+$ cells amongst splenocytes from 4-5 mice per treatment group, one-way ANOVA model with Tukey's correction.

(B) Values displayed are the means ± SEM of the frequency of naïve CD4 T cells ($CD4^+/CD62L^+/CD44^-$) amongst splenocytes from 4-5 mice per treatment group, one-way ANOVA model with Tukey's correction.

(C) Values displayed are the means ± SEM of the frequency of effector memory CD4 T cells ($CD4^+/CD62L^-/CD44^+$) amongst splenocytes from 4-5 mice per treatment group, one-way ANOVA model with Tukey's correction.

(D) Values displayed are the means ± SEM of the frequency of central memory CD4 T cells ($CD4^+/CD62L^+/CD44^+$) amongst splenocytes from 4-5 mice per treatment group, one-way ANOVA model with Tukey's correction.

(E) Representative flow gating.

Figure S16

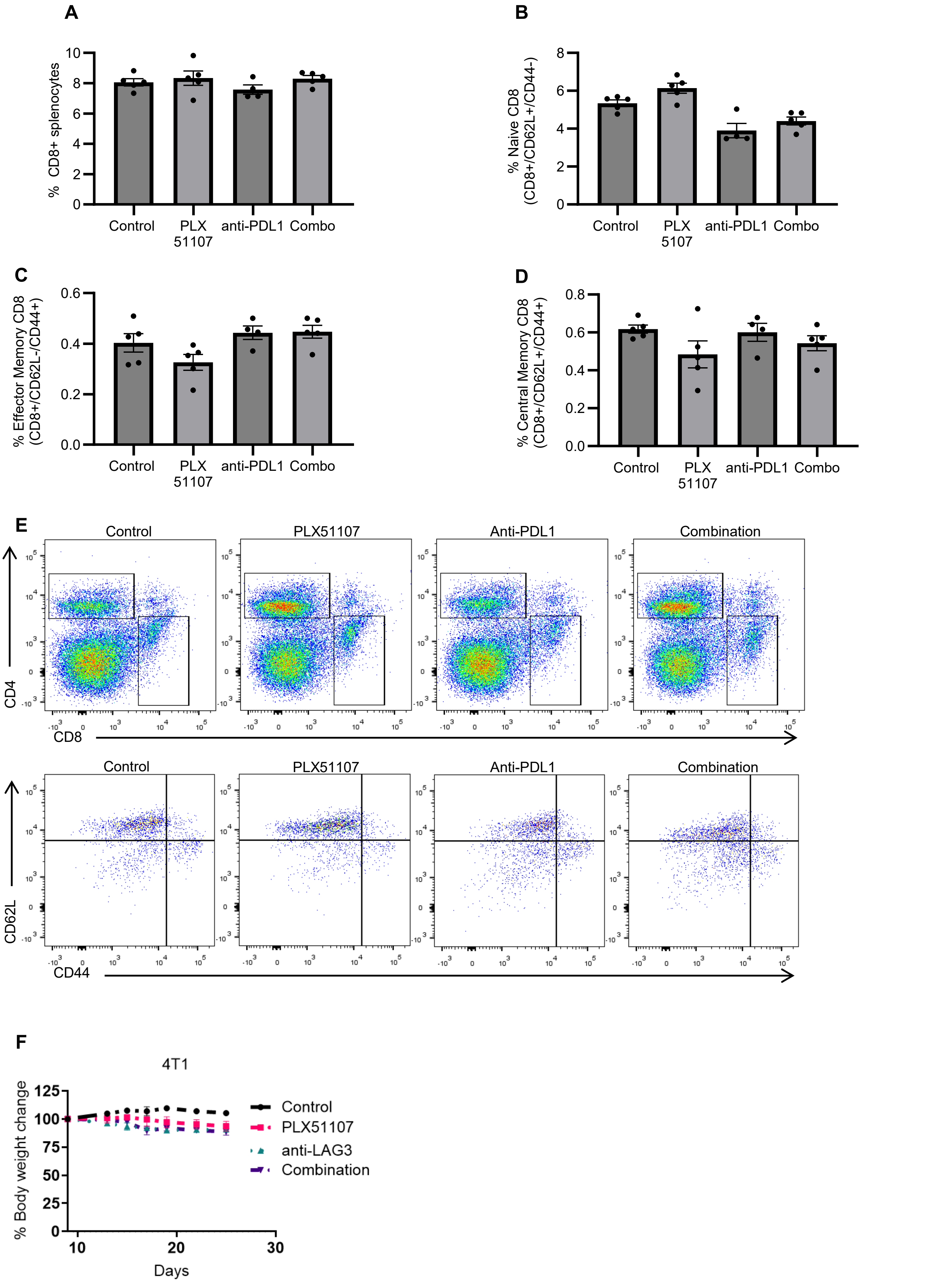

A
% CD8+ splenocytes
Control
PLX 51107
anti-PDL1
Combo
B
% Naive CD8 (CD8+/CD62L+/CD44-)
Control
PLX 5107
anti-PDL1
Combo
C
% Effector Memory CD8 (CD8+/CD62L-/CD44+)
Control
PLX 51107
anti-PDL1
Combo
D
% Central Memory CD8 (CD8+/CD62L+/CD44+)
Control
PLX 51107
anti-PDL1
Combo
E
Control
PLX51107
Anti-PDL1
Combination
CD4
CD8
CD62L
CD44
F
4T1
% Body weight change
Days
Control
PLX51107
anti-LAG3
Combination

Figure S16. Frequency of $CD8^+$ T cell phenotypes within the spleen in the 4T1 breast cancer model.

(A) Values displayed are the means ± SEM of the frequency of total $CD8^+$ cells amongst splenocytes from 4-5 mice per treatment group. $CD8^+$ cells were analyzed from the same splenocyte samples shown in Figure S15.

(B) Values displayed are the means ± SEM of the frequency of naïve $CD8^+$ T cells (CD4+/CD62L+/CD44-) amongst splenocytes from 4-5 mice per treatment group.

(C) Values displayed are the means ± SEM of the frequency of effector memory $CD8^+$ T cells ($CD8^+$/$CD62L^-$/$CD44^+$) amongst splenocytes from 4-5 mice per treatment group.

(D) Values displayed are the means ± SEM of the frequency of central memory $CD8^+$ T cells ($CD8^+$/$CD62L^+$/$CD44^+$) amongst splenocytes from 4-5 mice per treatment group.

(E) Representative flow gating.

(F) Body weights of 4T1-inoculated mice treated with control, PLX51107, or anti-LAG3 Ab as described in Figure 6. Values displayed are the means ± SEM.

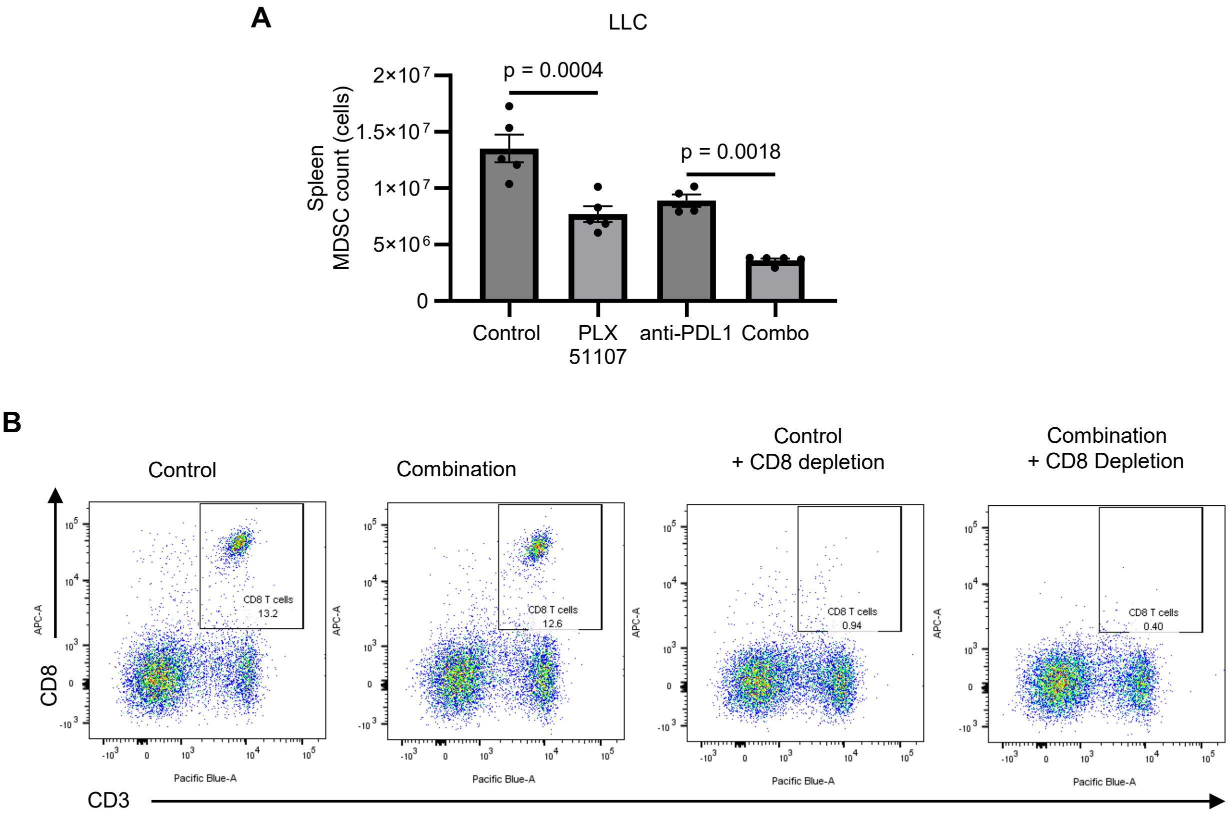

Figure S17.

(A) C57BL/6 were treated as in **Figure 6J**. Absolute MDSC counts were calculated by multiplying total cells within the spleen (counted using Z Series Coulter Counter) by the frequency of MDSC measured by flow cytometry. Bar graphs represent means ± SEM from 4-5 mice per treatment group, one-way ANOVA model with Tukey's correction.

(B) Representative flow plots of $CD3^+CD8^+$ double-positive cells in spleen from experiment **Figure 6M.**
