# Supplementary material for: BRD4 inhibition leads to MDSC apoptosis and enhances checkpoint blockade therapy
Source: J Clin Invest. 2025 Aug 5;135(19):e181975. doi: 10.1172/JCI181975 (PMC12483567; doi:10.1172/JCI181975)

Full Blots for Fig. 3C (Lanes used : 1,2)

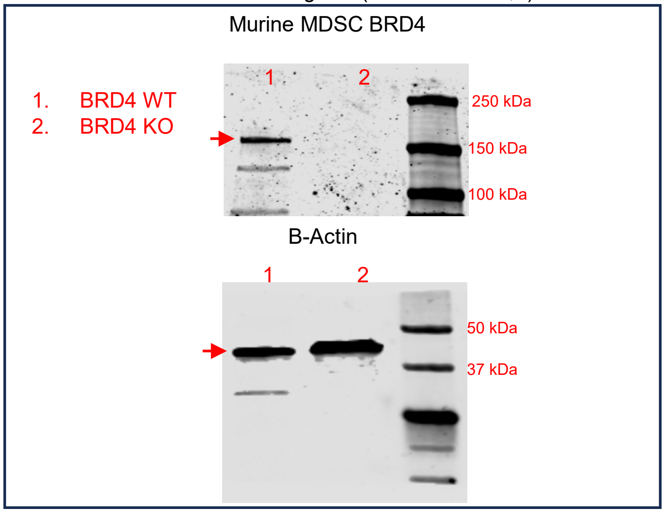

Full Blots for Fig. 5A (Lanes used: 1,2,3)

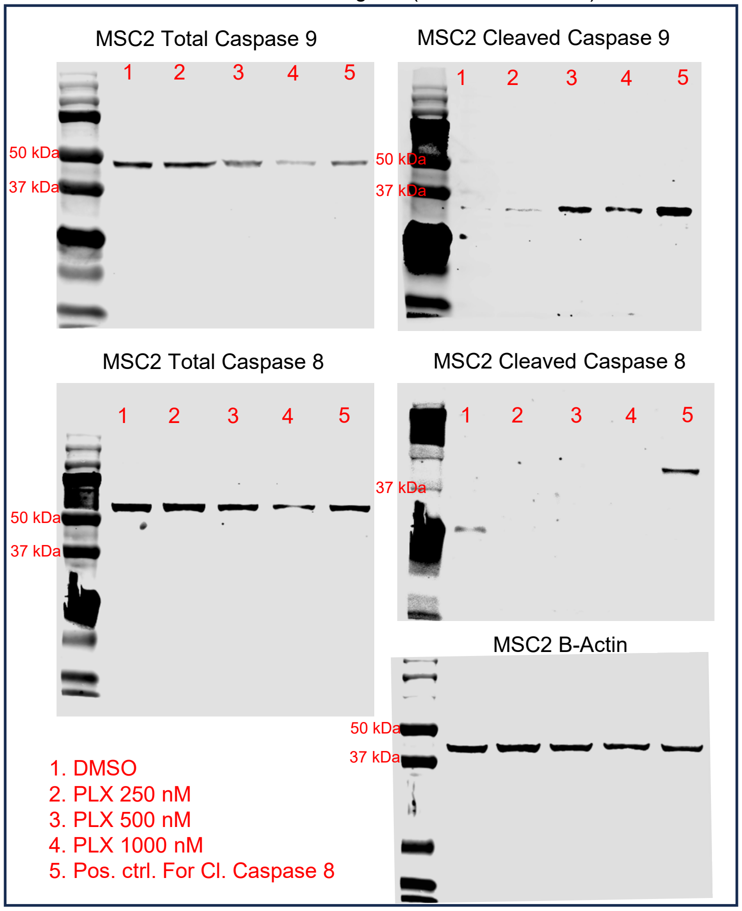

Full Blots for Fig. 5C (Lanes used: 1,2,3)

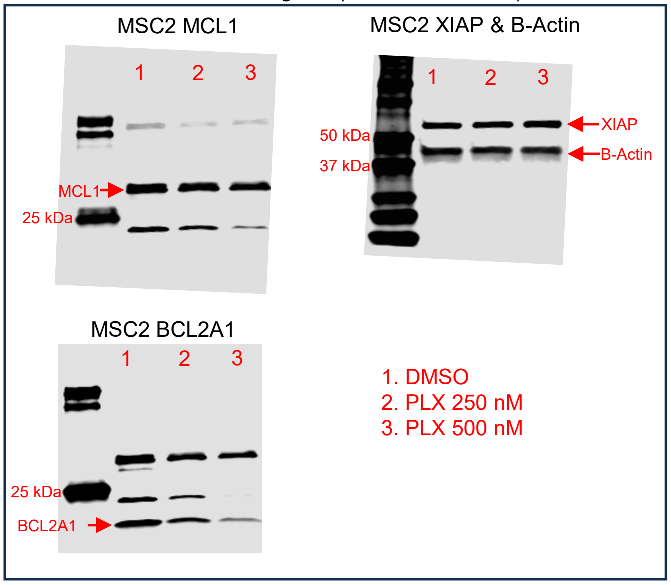

Full Blots for Fig. 5E (Lanes used: 1,2,3,4)

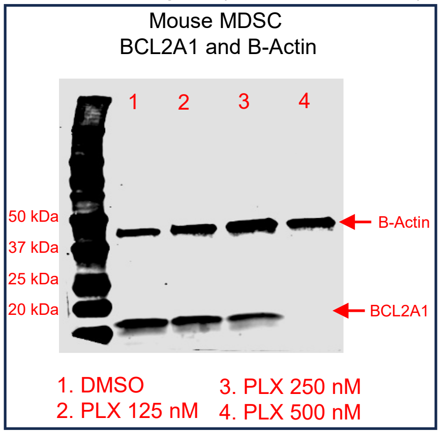

Full Blots for Fig. 5G (Lanes used: 1,2,3)

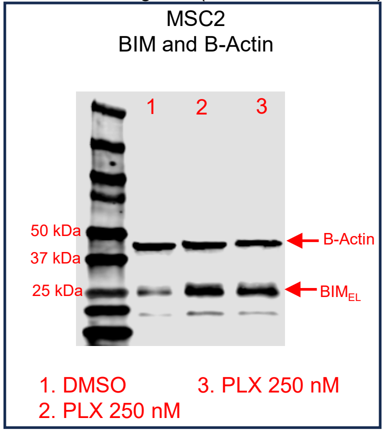

Full Blots for Fig. 5H (Lanes used: 1,2)

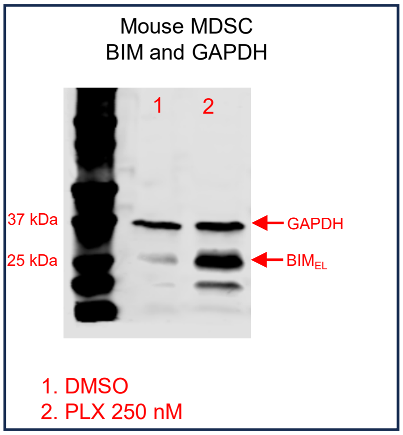

Supplement: Unedited blot and gel images [file jci-135-181975-s064.pdf]
